# A NEST-based framework unlocks massively parallel simulation of networks of multi-compartment neurons with customizable subcellular dynamics

**DOI:** 10.1038/s42003-026-11157-0

**Published:** 2026-10-27

**Authors:** Willem A. M. Wybo, Leander Ewert, Charl Linssen, Pooja Babu, Elena Pastorelli, Pier Stanislao Paolucci, Abigail Morrison

**Affiliations:** 1 Peter Grünberg Institute 15 – Neuromorphic Software Ecosystems (PGI-15), Forschungszentrum Jülich, Aachen, Germany; 2 Institute for Advanced Simulation (IAS-6), Computational and Systems Neuroscience, Forschungszentrum Jülich, Jülich, Germany; 3 Simulation and Data Lab Neuroscience, Jülich Supercomputer Centre, Institute for Advanced Simulation, Jülich-Aachen Research Alliance, Forschungszentrum Jülich, Jülich, Germany; 4 Software Engineering, Faculty of Computer Science, RWTH Aachen University, Aachen, Germany; 5 Istituto Nazionale di Fisica Nucleare, Sezione di Roma, Rome, Italy

**Keywords:** Biophysical models, Network models

## Abstract

While subcellular mechanisms in the dendrites of neurons govern the implementation of learning and memory in the brain, large-scale network simulations featuring such processes remain challenging to achieve. This can be attributed to a lack of appropriate software tools, as neuroscientific simulators focus on the one hand on highly detailed models, and on the other hand on massive networks featuring point neurons. Here, we fill this gap by implementing a framework for the massively parallel simulation of simplified dendrite models with customizable subcellular dynamics. We leverage the NEural Simulation Tool (NEST), the neuroscientific reference with respect to efficient massively parallel simulations of point-neuron networks. By co-opting the model description language NESTML, we generate C++ code implementing user-configurable subcellular dynamics. We show that the generated models run efficiently, leading to scalable NEST network simulations. To showcase relevant functionalities, we provide a proof-of-concept demonstration that a key sensory computation – the association of top-down context arriving at distal dendrites in layer 1 and feedforward sensory input arriving perisomatically – can be achieved in a single-shot fashion through apical calcium dynamics. Our work thus unlocks the study of how dendritic processes shape network function and learning at scale.

## Introduction

The brain computes in a massively parallel fashion. In the human brain, 86 billion neurons^1^ continuously convert synaptic inputs into action potential (AP) output signals. Moreover, even at the subcellular level, computations proceed in a massively parallel fashion. Approximately 150 trillion synapses^2^ (up to ~10,000 per neuron) are supported by complex molecular signaling networks within dendritic compartments. In itself, these signaling networks can also be understood as nanoscale computations that convert synaptic input, backpropagating APs, and local voltage and concentration signals into weight dynamics that support learning and memory^3^. Furthermore, the recent, explosive growth of AI technologies has shown the importance of scale^4^, as it was achieved in large part by applying a well-known attentional motif^5^ at massive scales^6–8^. Similarly, it can be expected that understanding the computational significance of subcellular processes will require embedding them in large-scale network simulations. It is only natural, thus, to use the parallelization and vectorization capabilities of modern supercomputing architectures to simulate the brain at scale in a massively parallel fashion.

Simulation of subcellular processes in biophysically detailed models has long been implemented in the successful NEURON simulator^9,10^, which is the most widely used tool for investigating models of individual neurons in great detail. Although NEURON admits efficient parallel simulations of network models through CoreNEURON^10^, users have to take care of the parallel distribution of neurons across MPI processes themselves^11^. Moreover, NEURON’s API is geared towards complex models, providing interfaces that read morphological data files, or interfaces that construct neurons as combinations of cylinders^9^; the parameters of individual compartments are not exposed. Recently, however, there has been a rise in interest in capturing dendritic computational principles in a manner that is as simple as possible, to explore the role of dendritic computation at a systems level^12–17^. Therefore, an intermediate level of description is necessary, where parameters of individual electrical compartments are exposed, so that customized neuron layouts featuring few compartments and abstract dendritic spike mechanisms can be created.

For such purposes, the NEural Simulation Tool (NEST) is a promising candidate as it targets massively parallel simulation of spiking network models^18^. Recent frameworks such as Arbor^19^ and EDEN^20^ illustrate the broader need for high-performance multi-compartment simulation tools, but address different workflow regimes: Arbor targets morphologically detailed multi-compartment networks on contemporary high-performance computing systems, whereas EDEN uses NeuroML-based model analysis and code generation to execute general neural models efficiently. In NEST, the simulation kernel has been optimized to efficiently communicate spike times across parallel processes^21–24^, and a key strength is the automatic distribution across MPI processes^25^ and OpenMP threads^26^ with little user overhead. NEST has recently been extended with a GPU-based framework for simulating spiking networks, further extending the capabilities for massive parallelism^24,27,28^. However, up until now, NEST has had limited options to simulate more detailed compartmental models as part of networks; users essentially had to develop custom C++ code, or had to resort to using NESTML to explicitly define compartmental equations. The first option, however, is cumbersome, while the second option is suboptimal, as NESTML was traditionally aimed at single-compartment models^29^. Standard NESTML will resort to an explicit Runge-Kutta integration scheme when compartmental dynamics are written explicitly^30^, whereas a semi-implicit cable-equation update solved with the Hines algorithm^31^ is more efficient and stable, and has been the gold standard for simulating spatially extended neuron models^9^.

To facilitate the parallel simulation of subcellular processes embedded in neurons that are themselves embedded in large networks, we have leveraged the NESTML model description language^30^ to develop a vectorized compartmental modeling framework in NEST. In this modeling framework, NESTML is used to describe dynamics that can, in principle, be simulated at each compartment of the neuron. In this sense, NESTML is thus used in a similar fashion to NEURON’s NMODL language^32,33^. These mechanisms can represent ion channels, synaptic receptors, concentration dynamics, integrate-and-fire type mechanisms, and arbitrarily complex synaptic plasticity rules. The mechanisms can then be embedded at runtime into a user-configurable compartmental layout, which already provides passive dynamics by default. The structure of the tree, representing the dendrite and/or axon, is also configured at runtime. To simulate the model, the voltage at the next time step is computed by the Hines algorithm, while the user-defined mechanisms are simulated in a vectorized fashion. This means that modern CPUs—e.g., supporting the AVX-512 instruction set—can compute intracellular dynamics for up to eight compartments in parallel^34^. Under the hood, the standard NEST machinery takes care of distributing the neurons across threads or MPI processes, and of transmitting spikes between them, resulting in distributed network simulations of compartmental models that are efficient and easy to implement. In this paper, we demonstrate the excellent scaling performance of the resulting network models. We also provide a detailed description of the coding principles in the Supporting Information (SI), demonstrating that such massive network simulations can be achieved with little overhead to the user.

To illustrate the key principles, we implement a network model incorporating the observation that top-down afferents target distal dendrites in layer 1 and cause dendritic calcium (Ca^2+^) spikes^35–38^. We capture this computation in a simplified, two-compartment neuron that approximates the interplay between dendritic Ca^2+^ spikes and somatic APs^16^. We then demonstrate that this interplay is necessary and sufficient to achieve single-shot association learning between top-down context and feedforward sensory input when peri-somatic learning is governed by spike-timing-dependent plasticity (STDP).

## Results

### Toolchain overview

Neuron models with complexity surpassing that of the point neuron are typically implemented using the multi-compartment (MC) modeling framework^39–42^, where compartments are connected to each other through coupling terms:

1 $$\underbrace{{c}^{i}\dfrac{d{v}^{i}}{dt} = {\sum}_{j\in {{{\mathcal{N}}}}^{i}}{g}_{C}^{ij}\left({v}^{j}-{v}^{i}\right)+{g}_{L}^{i}\left({e}_{L}^{i}-{v}^{i}\right)}_{{{\text{passive \; system}}}}+\underbrace{{\sum}_{c\in {{{\mathcal{C}}}}^{i}}{i}_{c}^{i}\left[{{{\bf{y}}}}_{c}^{i},{v}^{i}\right]+{\sum}_{r\in {{{\mathcal{R}}}}^{i}}{i}_{r}^{i}\left[{{{\bf{y}}}}_{r}^{i},{v}^{i},{S}_{r}^{i}\right]}_{{{\text{active \; currents}}}}.$$

Here *v*^*i*^ denotes the membrane potential in compartment *i*, *c*^*i*^ its capacitance, ${g}_{L}^{i}$ its leak conductance, and ${e}_{L}^{i}$ the leak reversal potential. The compartment *i* is coupled to its neighbors ${{{\mathcal{N}}}}^{i}$ through a coupling conductance ${g}_{C}^{ij}$. Due to the conservation of current, the coupling is symmetric, i.e., ${g}_{C}^{ij}={g}_{C}^{ji}$. By identifying the compartments with the nodes of a graph and the neighbor couplings with the edges, an MC model representing a dendrite is always a tree graph.

An a priori arbitrary set ${{{\mathcal{C}}}}^{i}$ of ion channels may be present in each compartment *i*, as well as an a priori arbitrary set ${{{\mathcal{R}}}}^{i}$ of synaptic receptors. Channel currents ${i}_{c}^{i}\,\left[{{{\bf{y}}}}_{c}^{i},{v}^{i}\right]$ typically depend on the local voltage, and also on an a priori arbitrary set of state variables ${{{\bf{y}}}}_{c}^{i}$. These state variables may simply describe channel opening based on the voltage, such as in the Hodgkin-Huxley formalism^43^, or may represent more complex intracellular dynamics^44–46^, such as a dependence on certain ionic concentrations^47^ or metabotropic interactions^48,49^. Compared to ion channels, receptor currents ${i}_{r}^{i}\,\left[{{{\bf{y}}}}_{r}^{i},{v}^{i},{S}_{r}^{i}\right]$ may similarly depend on complex intracellular dynamics through a set of state variables ${{{\bf{y}}}}_{r}^{i}$, to e.g., describe receptor dynamics and plasticity^50^, but have the additional feature that they integrate incoming trains of APs (${S}_{r}^{i}$).

**Fig. 1:**
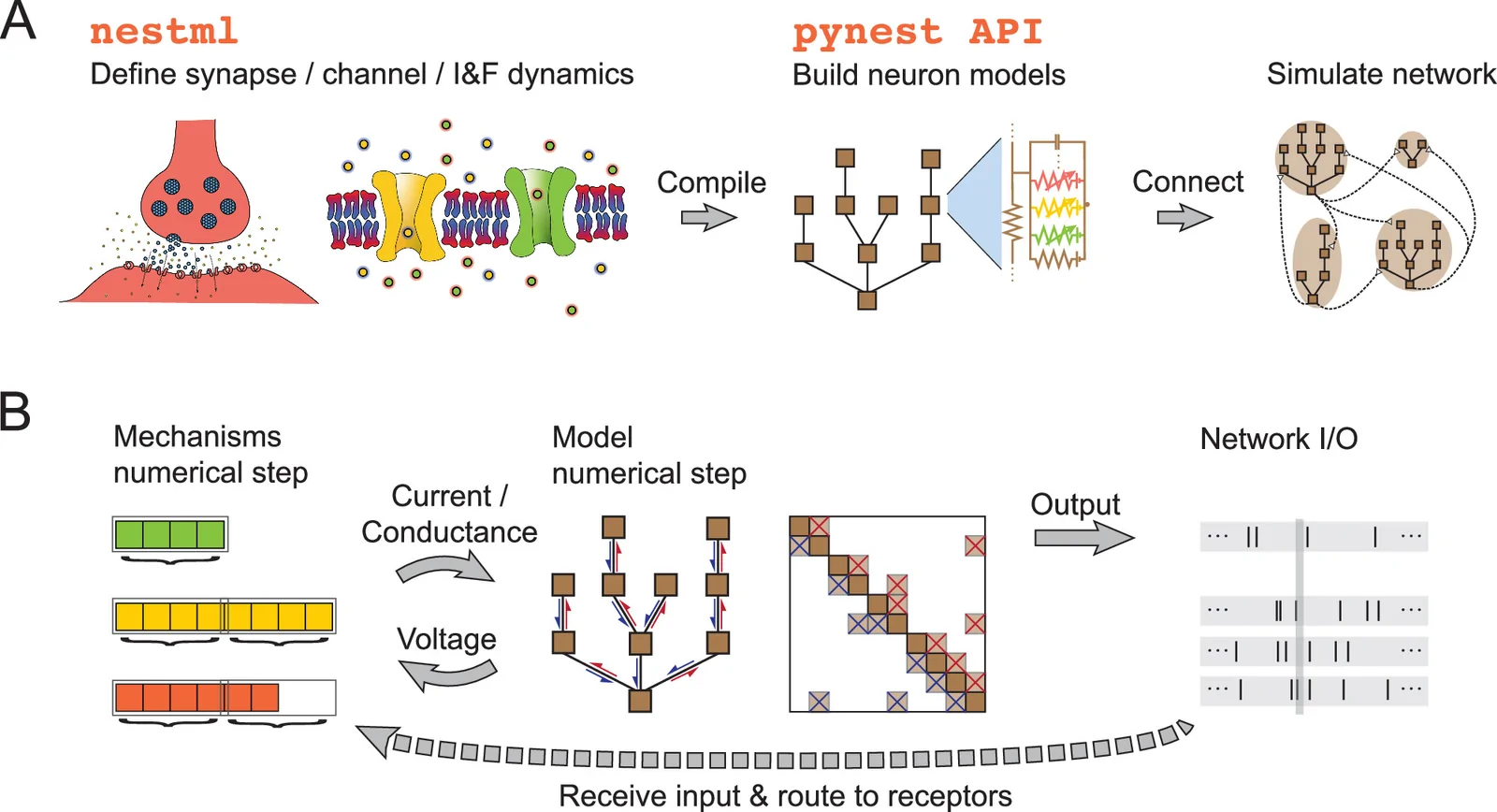
Overview of the compartmental simulation toolchain. **A** Dynamics of transmembrane currents, such as voltage-dependent ion channels or synaptic receptor channels, are defined in the NESTML modeling language (left). These dynamical models are then compiled into efficient C++ code to simulate them as part of a compartmental model, as in Eq. (1). The layout of this compartmental model is set in the PyNEST API (middle), including which channels and synapses are expressed in which compartments. Finally, this model is connected with other models to create a network simulation (right). **B** The internal layout of the model consists of *(i)* a vectorized membrane current integrator for every current type defined in NESTML (left), *(ii)* a tree graph structure to advance the compartmental model through the Hines algorithm (middle) and *(iii)* a network API. The membrane current integrator reads out the voltage and relevant concentration variables from the compartments for which the current is defined, and uses the CPU vectorization to integrate the currents for multiple compartments in parallel. The results of the integration are then provided as the currents/conductances required for the implicit Euler integration of the compartmental model. External inputs are connected to the model through specification of a receptor ID, referring to the index in the receptor list, which is then mapped internally to the correct mechanism.

Leveraging NEST and NESTML, we have built a vectorized framework for the massively parallel simulation of spiking neural networks, where the neurons are multi-compartment models that can be equipped with arbitrarily complex dynamics. These dynamics are described by continuously evolving ordinary differential equations (ODEs) as well as discrete spike-triggered updates. In this framework, active currents ${i}_{c}^{i}\,\left[{{{\bf{y}}}}_{c}^{i},{v}^{i}\right]$ and ${i}_{r}^{i}\,\left[{{{\bf{y}}}}_{r}^{i},{v}^{i},{S}_{r}^{i}\right]$ (cf. Eq. (1); Fig. 1A, left) are defined in NESTML, and compiled into SIMD vectorized C++ code (Fig. 1B, left), allowing modern CPUs supporting AVX-512 to integrate currents for up to eight compartments in parallel in double precision^34^. The passive system (cf. Eq. (1)) is assumed to always be present, and does not need to be described explicitly in NESTML. At runtime, after compiling the NESTML model, the dendrite structure is defined (Fig. 1A, middle) while building the model, at the same time also setting the parameters of both the passive and active currents. Internally, this layout is translated to a tree graph data structure, which is also used to perform the Hines update of the model (see “Methods”; Fig. 1B, middle)

Finally, networks are created by connecting presynaptic neuron populations to specific receptor ports of the postsynaptic neuron populations (Fig. 1A, right). The incoming spikes are then routed internally by the model to the appropriate receptor current (Fig. 1B, right).

### Single neuron benchmarks of the compartmental modeling framework

**Fig. 2:**
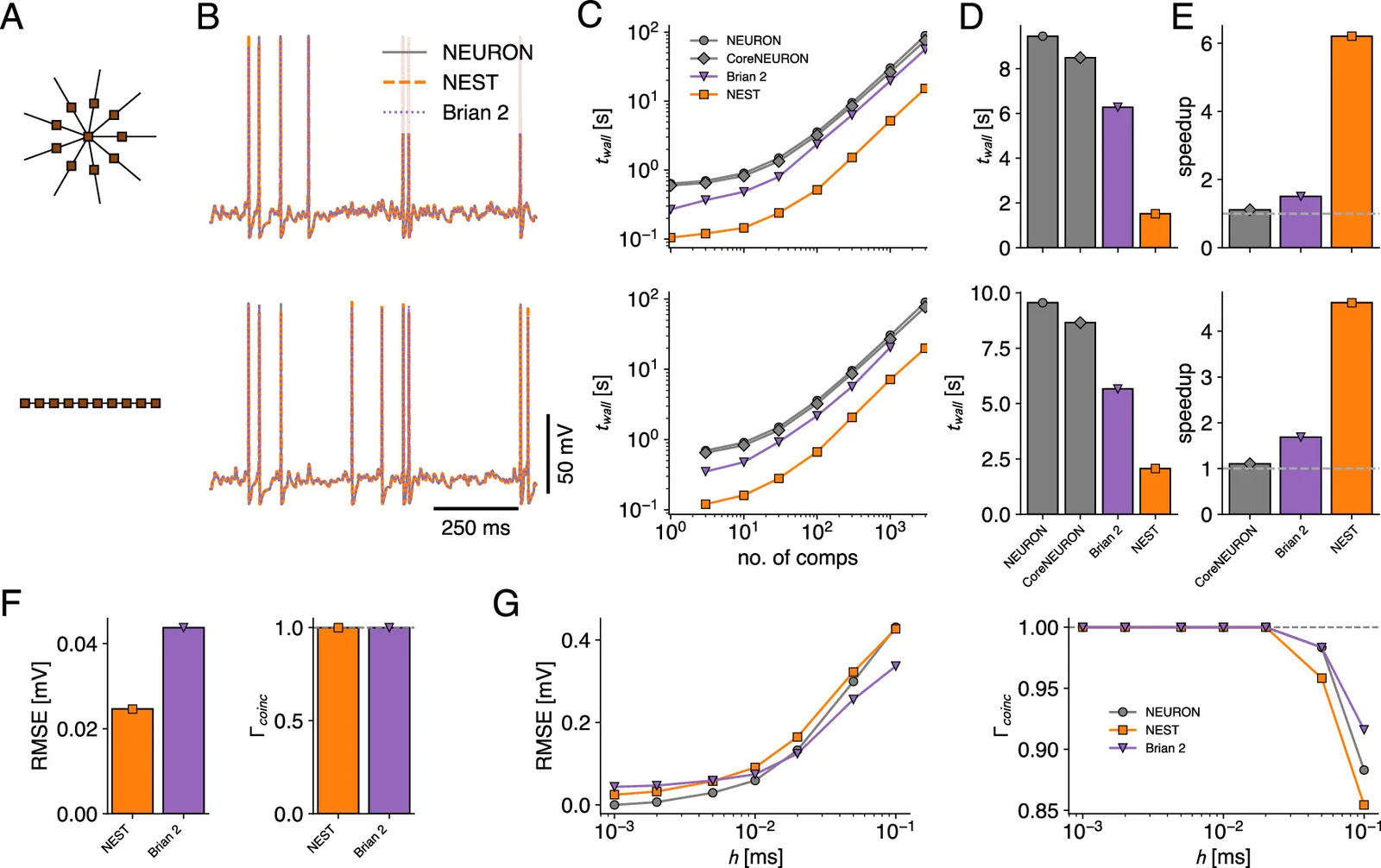
Performance and precision of the NEST-based compartmental modeling framework. **A** Two constructed morphologies allow precise control of compartment numbers and represent edge cases of the Hines algorithm: a star layout where each dendrite consists of a single compartment (top), resulting in a shallow and wide morphology tree, and a linear layout without branching (bottom), resulting in a deep but narrow tree. These morphologies are equipped with Na- and K-channels as well as an AMPA synapse at each compartment, and simulated in NEURON, Brian 2, and NEST. **B** Comparison of somatic voltage traces between NEURON, Brian, and NEST for the star morphology (top) and linear morphology (bottom), which both contain ten compartments in these simulations. **C** Wall clock time comparison between NEURON, CoreNEURON, Brian 2, and NEST for a simulation time of 10 s, as a function of the number of compartments for the star (top) and linear (bottom) morphology. **D** Detailed wall-clock time comparison for the 300-compartment case for each of the simulators. **E** Speedup factor of CoreNEURON, Brian 2, and NEST over standard NEURON. **F** Deviation of Brian 2 and NEST from NEURON reference trace, at a timestep of *h* = 0.1 ms using the star morphology with 10 compartments. Subthreshold root mean square error (RMSE) of the somatic voltage (left) and spike coincidence factor (*Γ*_coinc_) evaluated with a 5-ms window (right). **G** Precision of NEURON, Brian 2, and NEST as a function of the timestep (same neuron model as in **F**), where precision metrics are computed with respect to a NEURON reference simulation at *h* = 0.001 ms.

To test the efficiency of our generated code, we create two artificial morphologies where we can change the number of compartments at will. These morphologies also constitute two limit cases for the Hines algorithm: one of a fully branched model with one compartment per branch (Fig. 2A, top) and one of a fully linear dendrite with all compartments on a single branch (Fig. 2A, bottom). The former is wide and shallow, and the Hines inversion is efficiently vectorized by NEST’s solver, whereas the latter is narrow and deep, and the Hines inversion cannot be vectorized. Note that for the linear dendrite, the morphology itself remains the same while the different compartment numbers represent a different spatial discretization. For the branched model, the number of dendritic branches changes with the number of compartments, resulting in a higher effective leak and capacitance. While these effects do influence input integration, they are only of relevance for the network benchmarks, as the number of transmitted spikes may affect network runtimes but—in case of Hodgkin-Huxley type channels—does not influence single neuron runtimes. We equip all of the compartments with a sodium and potassium channel, and define identical models in NEURON, Brian 2, and NEST, as can be seen from a comparison of their membrane potentials (Fig. 2B). We then simulate these models for increasing numbers of compartments, and measure the runtime of a simulation of 10 s (Fig. 2C) on 2.25 GHz AMD EPYC processors that are part of the JURECA DC module^51^. The wall clock simulation time of NEST is significantly lower than the time of Brian 2, which in turn is lower than the time of NEURON and CoreNEURON for all tested numbers of compartments. Focusing on the 300-compartment case reveals speedup factors over NEURON of ~5–6 (Fig. 2D, E). To understand how the runtime of the model is distributed over the various computational steps, we profile the linear dendrite model (Fig. S1A), which reveals that solving the Hines algorithm takes up only a minor fraction of the runtime (up to 17%, Fig. S1B), whereas most of the time is spent updating the active mechanisms, and specifically computing the exponentials necessary for that purpose.

We test the precision of our NEST-based framework by comparing subthreshold voltage fluctuations and spike coincidence with a NEURON reference simulation at the same simulation time-step (0.1 ms, Fig. 2F), and do the same for the Brian 2 simulation. We observe that the NEST implementation is closer to NEURON in the subthreshold voltage, but all models reproduce the same spike train. To study precision as a function of time-step length, we simulate the model in all three simulators with time-steps varying from *h* = 0.001 ms to *h* = 0.1 ms (Fig. 2G), and take the NEURON implementation with *h* = 0.001 ms as reference. We find that all implementations exhibit very similar behavior. Remarkably, even though the Brian 2 implementation deviates most from NEURON at the same time-step length in the subthreshold voltage, it remains the most accurate at large time-steps.

**Fig. 3:**
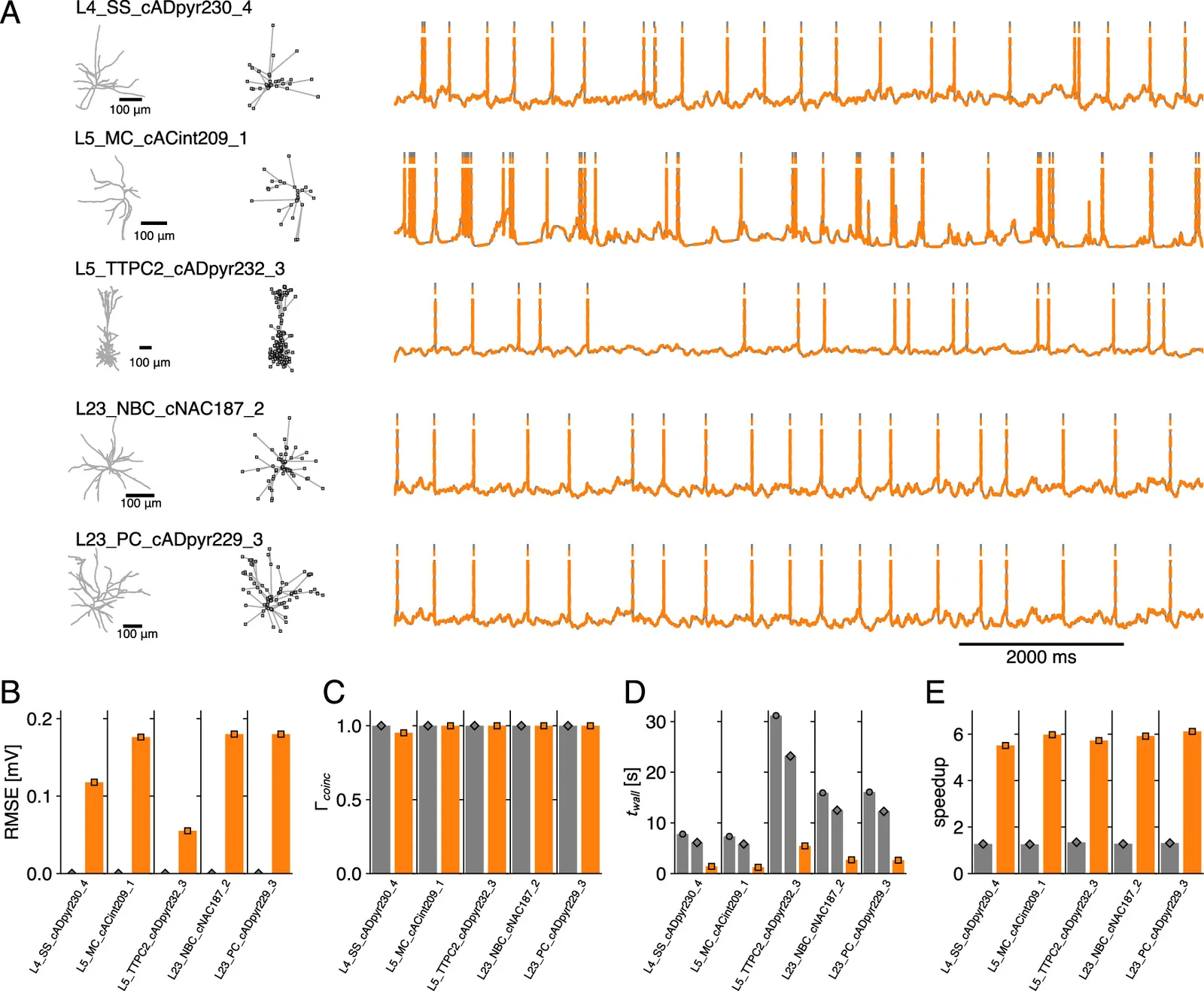
Evaluation of the NEST-based compartmental modeling framework on biophysically realistic neuron models. **A** Five exemplar neurons from the Blue Brain Project database^53^ were embedded in NEAT^52^ and simplified by retaining leafs, soma, and all bifurcation sites, following Wybo et al.^81^. The resulting models were equipped with an AMPA+NMDA and GABA receptor at each compartment and stimulated with random input spike trains (identical in NEURON and NEST). Somatic responses for NEURON (gray) and standard NEST (orange) on the right. **B** RMSE of the subthreshold somatic voltage for each neuron model simulated with CoreNEURON and NEST, with respect to standard NEURON as reference (same markers as in Fig. 2). **C** Same as in (**B**), but for the spike coincidence factor *Γ*_coinc_. **D** Wall time for a 10 s simulation of each neuron model, comparing NEURON, CoreNEURON, and NEST (simulator-specific markers as in Fig. 2C). **E** Speedup of CoreNEURON and NEST compared to standard NEURON.

Code efficiency and accuracy depend in a non-trivial manner on model layout. To assess whether our findings from the artificial morphologies with limited channel mechanisms generalize to a biologically plausible model range, we used NEAT^52^ to implement five exemplar models from the Blue Brain Project (BBP) library^53^ in NEURON and NEST (Fig. 3A). These models are based on morphological reconstructions and contain a range of voltage-dependent Na^+^-, K^+^-, and Ca^2+^-channels, as well as Ca^2+^ concentration dynamics and a Ca^2+^-activated K^+^ channel. We stimulate these models with identical spike trains consisting of random Poisson trains superimposed over Poisson-distributed spike bursts, which target AMPA+NMDA and GABA synapses in each dendritic compartment. We found that both the subthreshold voltage metric (Fig. 3B) and the spike coincidence metric (Fig. 3C) showed strong agreement between NEURON and NEST. Measuring the wall clock runtimes (Fig. 3D), we found speedup factors over NEURON of ~6 (Fig. 3E), convergent with the results on the artificial morphologies. One limitation of our comparison is that the BBP models only feature Hodgkin-Huxley type channels, and therefore we could not directly test multi-state channel or receptor kinetics. Even though fully supported by the NESTML code generation pipeline, NEAT lacks support for multi-state kinetics. Therefore, it would be challenging to compare identical models in NEURON and NEST.

Finally, the analytical algorithms to compute impulse response kernels available in NEAT^52^ enabled us to test accuracy against analytical spatio-temporal solutions. To this end, we used the BBP L5_TTPC2 morphology and instantiated the cell with a representative passive physiology (specific capacitance, axial resistance, and membrane conductance taken from Major et al.^54^ and spine correction from Rhodes et al.^55^). We then analytically computed impulse response kernels using NEAT for the full cylinder model of the cell at five locations (Fig. S2A). Next, we discretized the morphology spatially using NEAT, and exported the obtained model to both NEST and NEURON, allowing us to simulate the same response kernels with both simulators (Fig. S2B). We then computed the RMSE of the simulated kernels with respect to the analytical solution, and found a near-perfect match between both simulators as well as good convergence for decreasing time- and distance-steps (Fig. S2C). This analytical validation confirms solver correctness in the passive regime, where a closed-form reference exists. For models with active conductances, there is no such reference, so agreement is instead assessed via cross-simulator comparison (Figs. 2 and 3). Discretization errors due to the first-order linearization (cf. Eq. (2)) were found to be consistent across simulators and in line with the expectation that they are largest during fast non-linear transients such as the spike upstroke.

### Scalable network simulations with morphologically extended neurons

**Fig. 4:**
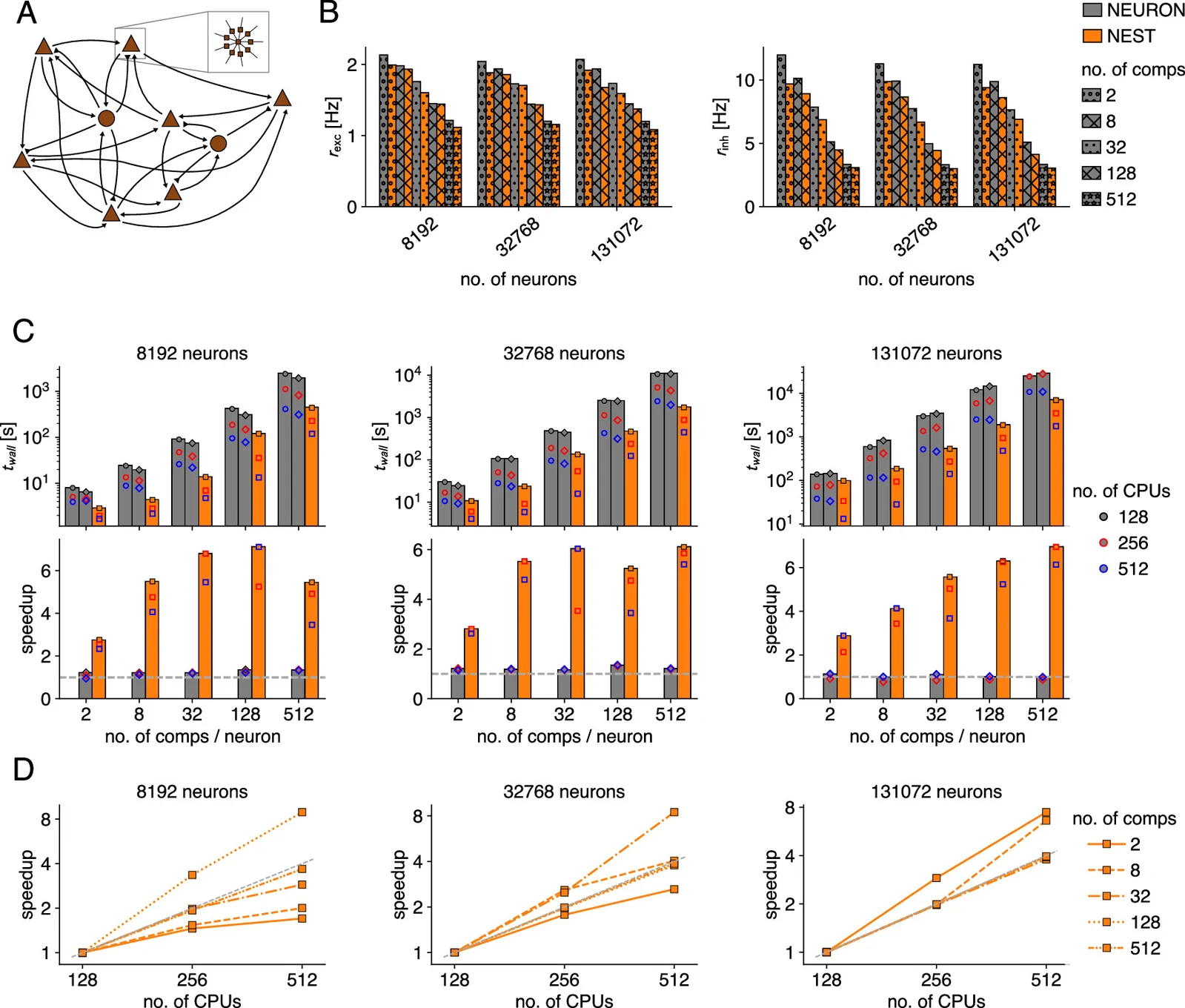
A systematic network scaling benchmark for massively parallel simulations of networks with morphologically extended neurons. **A** Using the star morphology introduced earlier (Fig. 2), we develop a network benchmark in NEURON and NEST where the number of dendritic compartments as well as the number of neurons can be tuned at will. Of the total number of neurons, 80% are excitatory (triangle) and 20% inhibitory (sphere), and each population connects randomly but with a fixed in-degree to itself and to the other populations, resulting in connectivity that, in a distributed simulation, spans across all MPI processes and threads equally. To drive the network, each neuron receives external input in the form of an independent Poisson spike train. **B** Resulting firing rates in the network are within physiological ranges, match closely for the NEURON and NEST implementations, are independent of the number of neurons because of the fixed in-degree, and depend only weakly on the number of compartments. **C** Wall clock times (top) for 10 s simulations of 2^13^ (left), 2^15^ (middle), and 2^17^ (right) neurons, for—from left to right (marker shapes as in Fig. 2)—NEURON, CoreNEURON, NEST; bottom shows speedup compared to standard NEURON. Numbers of CPU cores over which the network is distributed are indicated with marker edge color. **D** Strong scaling performance of the NEST network models, i.e., speed-up measured in wall clock runtime when the same network is distributed over increasing amounts of CPU cores, for a fixed simulation time of 10 s. Panels show an increasing number of neurons (left 2^13^, middle 2^15^, and right 2^17^), with in each panel different compartment numbers per neuron (2, 8, 32, 128, and 512). The dashed gray line shows the case where increasing the number of CPU cores by a given factor results in an equal speed-up.

We furthermore test the scaling performance of a recurrently connected spiking network, in which the neurons have the star morphology (Fig. 2A). Networks are simulated with increasing numbers of neurons (2^13^, 2^15^, 2^17^), and for each network size we probe five different numbers of compartments (2, 8, 32, 128, and 512). Of the total number of neurons, 20% of neurons are inhibitory, and the other 80% are excitatory. To minimize network size effects, we randomly connect these neurons with a fixed in-degree, and also connect independent Poisson spike generators in a one-to-one fashion to the excitatory population. Note that the dendritic compartment receiving the connection is also chosen at random. The fixed in-degree ensures that the firing rates do not differ across network sizes (Fig. 4B). Further, the random connectivity ensures that afferent connections can originate from any of the neurons in the network, independent of the MPI process or threads in which the neurons end up. By consequence, this is an excellent layout to test scalability. Furthermore, we apply a weight normalization based on the input resistance, to limit the effect of neuron size on output firing rate, induced by varying the number of compartments in the star layout. This results in firing rates within biophysical ranges, whose variation for different compartment numbers was sufficiently low so as not to critically affect runtime benchmarks (Fig. 4B). We then measure the wall clock runtimes of network simulations of 10 s, while distributing them over increasing numbers of CPUs (Fig. 4C), and observe similar speedups over NEURON as before. Furthermore, the speed-up as a function of the number of CPUs for fixed network and neuron size is close to the theoretical optimum for strong scaling experiments (Fig. 4D). This excellent scaling performance was observed for all network sizes, with as few as 16 neurons per CPU, indicating that large-scale networks can be distributed over many CPUs with little overhead. We note that achieving these distributed network simulations in NEST is straightforward and requires only a few lines of code, as NEST natively takes care of distributing the simulation across MPI processes and/or OpenMP threads.

### Hybrid few-compartment models uniting detailed and abstract dynamics

**Fig. 5:**
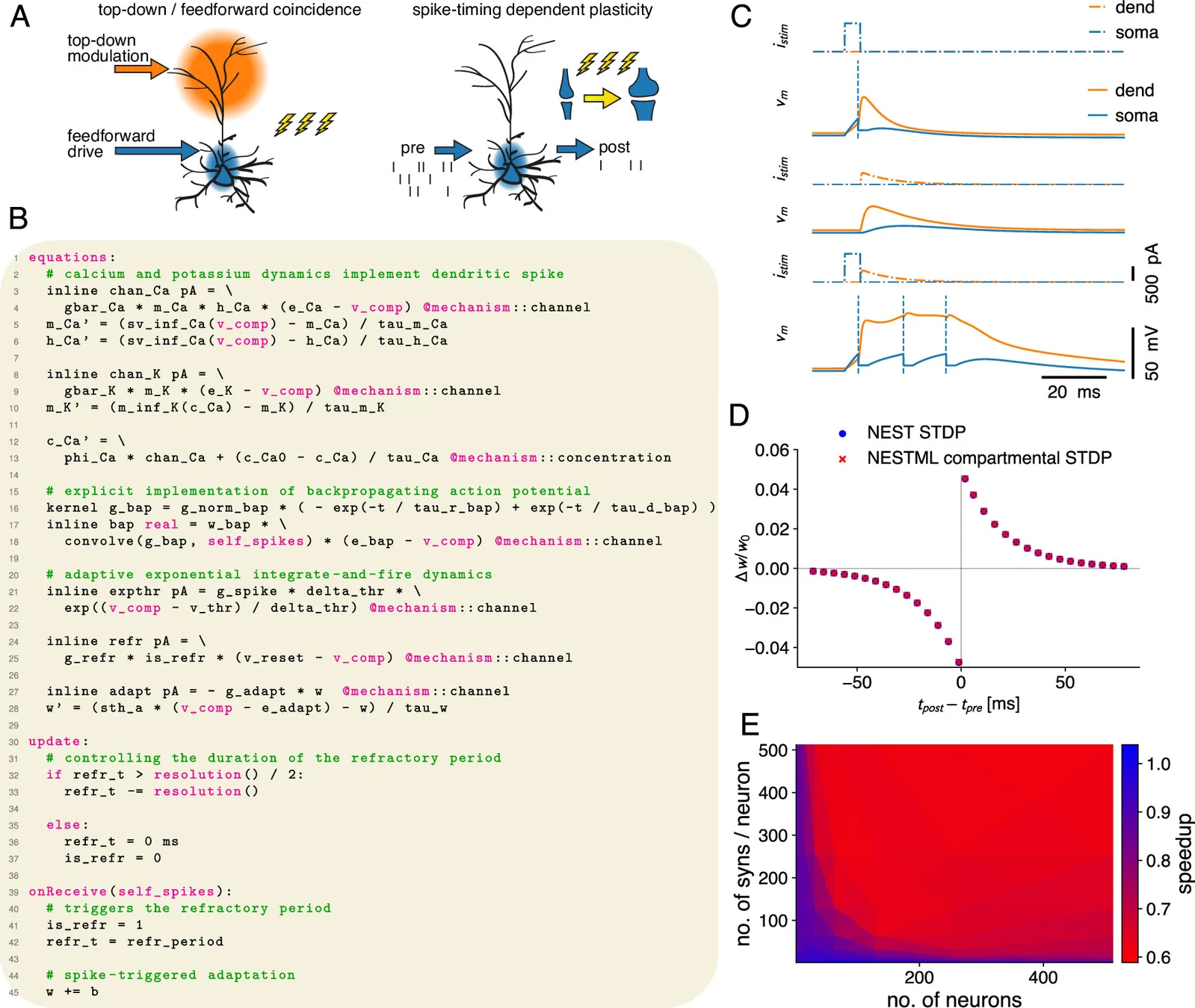
Exemplar implementation of a hybrid model uniting dendritic calcium dynamics and a somatic adaptive exponential integrate-and-fire mechanism. **A** Coincidence of apical top-down input and peri-somatic feedforward input elicits BAC-firing, a calcium-mediated coincidence detection mechanism (left). In a two-compartment NESTML model, this is combined with somatic STDP (right). **B** NESTML code excerpt of the Ca-AdEx model, which features the Ca^2+^-dynamics necessary for BAC firing, as well as AdEx dynamics for somatic spike generation. Note that the NESTML blocks specifying parameters and state variables, as well as the NESTML model implementing STDP, are omitted for clarity. SI and Figs. S4–S7 provide a more complete overview of the essential code concepts. **C** Reproduction of the BAC-firing protocol with the two-compartment Ca-AdEx model, where the model spikes once in response to a somatic stimulus (top), does not spike in response to a dendritic stimulus (middle), but fires a burst of APs in response to the coincidence of both (bottom). Somatic and dendritic input currents (blue resp. orange) are shown above the somatic and dendritic response traces (blue resp. orange). **D** Reproduction of the STDP window with the two-compartment Ca-AdEx model, once using the standard NEST STDP mechanism (blue dot) and once using the NESTML one embedded in the MC framework (red cross). **E** Speedup factor of the NESTML STDP mechanism over the standard NEST STDP mechanism.

A major use-case for our simulation framework is defining hybrid MC models that combine abstract integrate-and-fire dynamics representing AP and/or dendritic spike generation with select biophysical processes of interest (Fig. 5A). Through the NESTML description language^30^, the equations governing the dynamics of these models can be defined compactly (Fig. 5B), while a scalable network simulation can then be achieved easily with NEST, as argued in the previous section. We illustrate these principles in a model uniting adaptive exponential (AdEx) integrate-and-fire dynamics^56^ and dendritic calcium dynamics (Ca-AdEx), which we base on our prior work^16^. These calcium dynamics, which drive Ca^2+^-spikes, couple top-down inputs in the distal apical dendrites of pyramidal neurons to the feedforward inputs impinging peri-somatically^35–37,57^ (Fig. 5A, left). The resulting Ca^2+^-spikes are implicated in computations ranging from error backpropagation to context adaptation^58–62^, and require a calcium-mediated interplay between calcium channels and calcium-activated potassium channels, next to backpropagating APs supported by dendritic sodium channels^63^. In this hybrid, two-compartment description, we cannot support backpropagating APs in the same way that biophysically detailed models do, where the apical dendrite is modeled through a multitude of compartments. Instead, backpropagation of APs to the dendritic compartment is incorporated explicitly (Fig. 5B, lines 16-18). We test this model by reproducing the backpropagation-activated coincidence detection protocol from Hay et al.^63^ (BAC-firing), where the model spikes once in response to a somatic stimulus, does not spike in response to a dendritic stimulus, but fires a burst of APs in response to the coincidence of both (Figs. 5C and S6).

While static weights permit the study of dynamical network states, understanding how biological brains learn requires the investigation of how neural activity and neurotransmitter signals drive weight changes. NEST provides a wide variety of plasticity rules for this purpose, which range from classic STDP^64,65^ to credit assignment rules for error-driven learning^66^. These plasticity rules can straightforwardly be combined with the MC modeling framework (Fig. 5A, right, Fig. S7), where several options are available to the user. For instance, NEST’s built-in plasticity rules can be combined with any NEST model, including the compartmental ones. Alternatively, the MC framework also facilitates the definition of custom plasticity rules in NESTML, which can read out any compartmental state variables. We demonstrate this functionality by re-implementing the STDP rule in the MC framework and combining it with the Ca-AdEx model (see SI), which results in dynamics equivalent to the standard NEST STDP implementation (Fig. 5D). Note that the standard NEST plasticity mechanisms leverage the fact that the STDP mechanics do not depend in a continuous way on any state variables, and therefore perform irregular updates only when pre-spikes arrive. The NESTML plasticity implementation does not make this assumption, as we orient our implementation towards cases where plasticity mechanisms depend on local dendritic state variables^50^. This results in a moderate decrease in computational efficiency for the STDP mechanism (Fig. 5E). However, for mechanisms depending on local dendritic dynamics, vectorization and memory locality benefits are more likely to outweigh this.

### Leveraging dendritic dynamics for single-shot learning

While a broad consensus is emerging that dendritic dynamics fundamentally shape brain functions involving learning and memory^61,62,67–72^, large-scale network studies of this are rare because of a lack of appropriate simulation tools. Our NESTML compartmental modeling framework allows us to straightforwardly implement network simulations featuring dendritic dynamics, which we demonstrate by embedding the previously described neuron reproducing dendritic Ca^2+^-spikes in a network model. One striking aspect of the cortical layout is that contextual afferents from higher cortical areas target layer 1^73^, where they connect to distal apical dendrites of pyramidal cells and disinhibitory vasoactive intestinal peptide-expressing (VIP) interneurons^74^. Together, this allows for the generation of apical Ca^2+^-spikes, which fundamentally shape perception^38,75^.

**Fig. 6:**
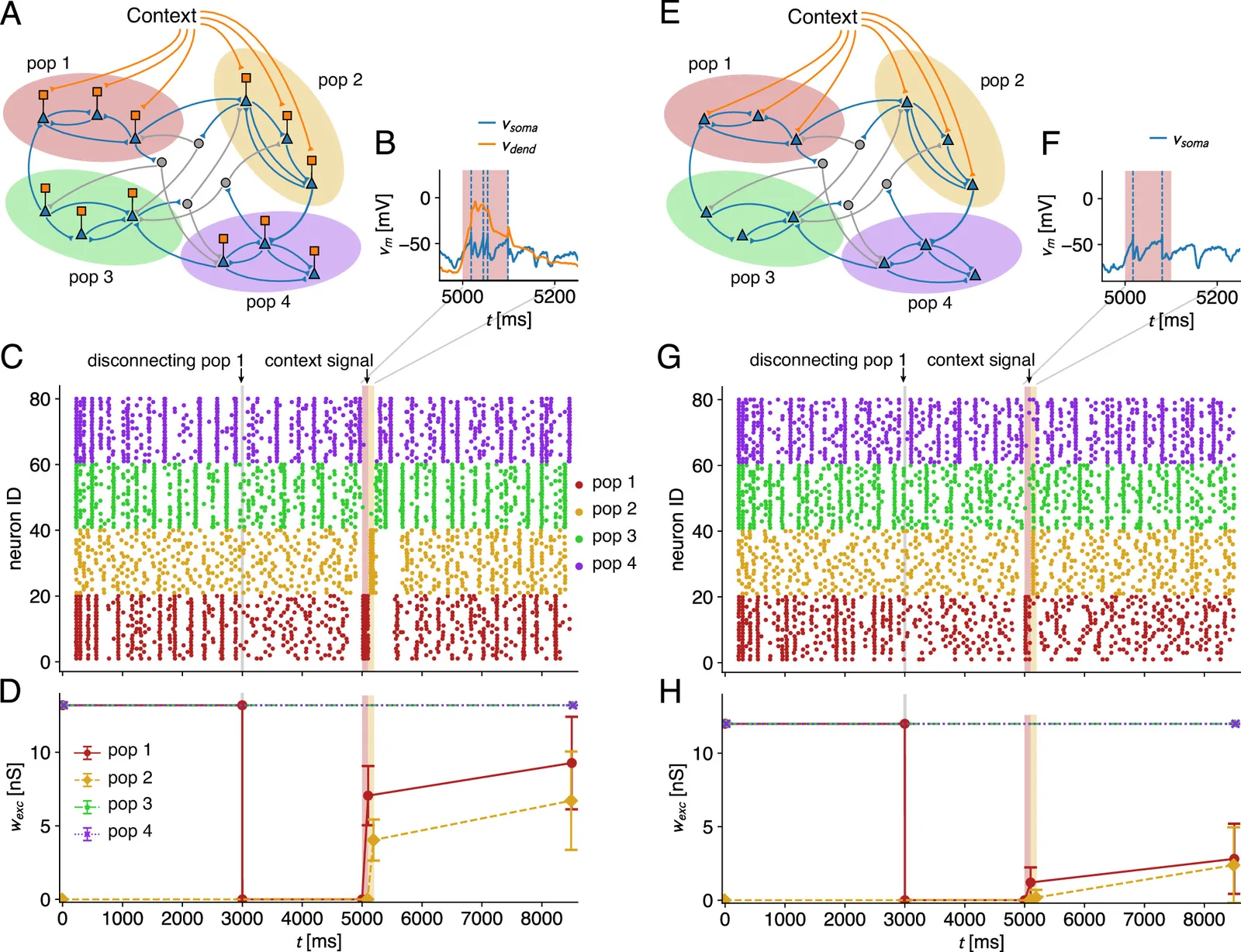
Dendritic, but not somatic, contextual input drives single-shot association learning to yield a winner-take-all network model. **A** A network with four populations of Ca-AdEx neurons is connected recurrently through somatic connections, while inhibitory interneurons implement broad inhibition that results, for strong intra-population excitatory weights, in a winner-take-all network where each population fires in unison and inhibits all others. Contextual connections impinge on the dendritic compartment. **B** The contextual input triggers a dendritic Ca^2+^-spike, resulting in somatic burst firing. **C** Raster plot of the spiking network activity. Initially, populations 1, 3, and 4 have high intra-population connection weights and therefore each firing in a concerted manner, while population 2 has zero connection weight and fires in an uncoordinated fashion. To illustrate the effect more clearly, connection weight in population 1 is set to zero at *t* = 3000 ms, resulting in random firing. A 25 ms context signal, in the form of Poisson input, is then delivered first to population 1 at *t* = 5000 ms, and then to population 2 at *t* = 5100 ms, while STDP is turned on in the intra-population connections of populations 1 and 2 at *t* = 5000 ms. **D** Corresponding intra-population weights in the network. **E**–**H** Same as (**A**–**D**), but for point neurons, where the context signal has to target the somatic compartment.

Here we demonstrate, in a recurrently connected network consisting of the Ca-AdEx neurons, that contextual input impinging on the apical dendrite, in coincidence with a peri-somatic stimulus, can drive the formation of experience-specific neural assemblies in a single-shot fashion, following an apical stimulus lasting only 25 ms. To this purpose, we customize a toy model that supports the description of a network composed of assemblies of neurons engaged in a winner-take-all dynamic, capable of incremental learning and of entering different brain states (*WTA-States*, cf. “Methods”). Thus, in this set-up, apical input enables learning of individual experiences at a rate compatible with the fastest conscious biological perceptions^76,77^. We construct a network model with four assemblies of excitatory neurons, where inhibitory interneurons implement a winner-take-all dynamic (Fig. 6A). With recurrent connections impinging perisomatically, neurons belonging to an assembly fire in unison, inhibiting all other assemblies (Fig. 6C). Conversely, weak intra-cluster connections lead to random firing, as is exemplified by setting the intra-assembly connection weights for population 1 to zero at *t* = 3000 s (Fig. 6D). We then target the apical compartments in the randomly firing clusters (populations 1 and 2) with excitatory contextual inputs. To mimic VIP-mediated disinhibition, we provide inhibitory inputs to the apical compartment in the 200 ms preceding the excitatory input, so that the release from inhibition occurs when the excitatory input arrives. Together, this elicits a Ca^2+^-spike in the targeted populations (Fig. 6B), in turn triggering burst firing in the associated neurons. In combination with STDP plasticity on the intra-assembly connections, this burst leads to rapid potentiation of these synapses (Fig. 6D) and restores coordinated ensemble firing (Fig. 6C). In response to an apical 25 ms contextual signal, a first ensemble is formed in 100 ms (red population in the network supporting apical amplification, Fig. 6C, D; start time of learning at 5000 ms), immediately followed by the formation of another assembly (yellow population, start time 5100 ms, same panels). Thus, in this network model, the Ca^2+^-driven apical compartment enables formation of new memories at a rate of ~10 Hz.

When the network consists of point neurons (Fig. 6E), the same context signal as before has to impinge perisomatically, and hence no burst firing is elicited (Fig. 6F). As a consequence, the context signal does not drive potentiation and leads to a failure to obtain ensemble formation in a single-shot fashion (Fig. 6G, H). Therefore, in neurons lacking apical amplification mechanisms, contextual stimulation of much longer duration would be required, resulting in a substantially lower rate of memory formation. In many studies so far, only such point-neuron implementations were investigated, as only these were available for straightforward large-scale simulation. Our results therefore also highlight that mechanistic conclusions about brain computation obtained from point-neuron network simulations should be treated with care.

## Discussion

In this work, we have presented a novel framework for creating large-scale network simulations consisting of multi-compartment neuron models, leveraging the spiking neural network simulator NEST^78^. This framework is based on the existing model description language NESTML^29,30,79,80^, exploiting its syntax features to describe membrane currents and concentration dynamics. We have also extended NESTML with a new code generation pathway that builds efficient multi-compartment C++ code based on the provided equations. The models can then be configured in the PyNEST API and connected in networks to be simulated using the standard NEST routines. Through the use of a structure-of-arrays layout, the models are efficiently vectorized, leading to considerable single-neuron speedups with respect to the NEURON simulator. Together with the native MPI support for massively parallel network simulations built into NEST, this results in a framework that allows the specification and simulation of networks with dendritic dynamics at scale. It should be noted, however, that at present NEST’s neuron distribution across CPUs does not account for differences in model complexity, so simulations with strongly heterogeneous cell types are not yet explicitly load balanced. Nevertheless, for large networks this limitation will often be mitigated in practice, as the distribution of many cells across CPUs is likely to yield an approximately balanced computational load on average.

We compared runtimes across a broad range of benchmarks—from biophysically realistic neurons to large-scale recurrent networks—with NEURON and CoreNEURON, and for the single-neuron artificial morphology benchmark also with Brian 2’s SpatialNeuron implementation. Dendrify^14^ provides a complementary Brian 2-based framework for constructing reduced dendritic spiking neuron models through a simple user-facing API. However, Dendrify builds on standard Brian 2 NeuronGroups rather than SpatialNeuron, and therefore uses explicit forward integration instead of a Hines-style implicit solver for arbitrary multi-compartment tree morphologies. For electrically coupled compartmental trees, such schemes are only conditionally stable and may require prohibitively small timesteps as axial coupling strength, branching complexity, or spatial discretization increase. This is precisely the regime for which Hines-type solvers are generally used. For this reason, the relevant Brian 2 baseline for the benchmark class considered here is SpatialNeuron, whereas Dendrify addresses a complementary reduced-model use case. Finally, NEURON offers a range of solvers, including higher-order methods such as Crank-Nicholson or variable time-step methods. While these methods are useful to obtain high-accuracy single-neuron solutions, they come with considerable drawbacks in large-scale distributed network setups (e.g., Crank-Nicholson occasionally induces oscillations that lead to spurious spikes, whereas variable time-step methods substantially slow down the network simulation due to variable update times). Therefore, we follow the same strategy as CoreNEURON and focus our numerical implementation on the efficient and stable implicit Euler algorithm.

Although there are no a priori restrictions on the amount of detail and the number of compartments a model can have, the model configuration interface in the PyNEST API has deliberately been kept low-level, i.e., the parameters for the individual compartments have to be explicitly provided by the user. This interface is hence well-suited to multi-compartment models containing few compartments, which are increasingly used to capture key dendritic computations in a minimal fashion^12–17^. However, the NEST initiative has also recently been extended with the NEural Analysis Toolkit (NEAT)^52,81^, a novel tool that allows the researcher to define morphologically detailed neuron models, analyze them, and create simplifications thereof. These fully detailed and simplified neuron models can then be exported to NEURON and NEST, which we demonstrate here with models from the BBP library^53^. To achieve the latter, NEAT leverages the same NESTML-based code-generation pipeline and C++ framework as described here, resulting in the efficient simulation of networks of biophysically detailed neuron models. By consequence, the framework is already usable for a broad range of modeling tasks, provided that users work at the appropriate level of the software stack. Simple few-compartment models can be constructed directly in the PyNEST API by explicitly specifying compartments, receptors, and connectivity. As the pipeline co-opts pre-existing NESTML syntax features, it is already applicable to a broad range of use cases without requiring changes to the underlying code-generation, as we illustrate with our Ca-AdEx model (Fig. 5). For workflows involving realistic morphologies, model simplification, or cross-simulator export, NEAT provides a higher-level interface that leverages the same NESTML-based generation pathway used here, but is not required for simple hand-built models. We have leveraged this workflow to design cross-simulator benchmarks (Figs. 2–4). New users are furthermore supported through the NEST GitHub issue tracker, the NEST user mailing list, regular hackathons, and tutorial and workshop sessions at neuroscience conferences.

By enabling fine-grained parallelization and vectorization, GPUs are highly adept at implementing computations requiring many identical operations^82^. This renders them ideally suited for, e.g., matrix multiplication, leading to large speed-ups for simulating artificial neural networks compared to normal CPUs. A key reason is that dense matrix multiplication has a high arithmetic intensity, i.e., each block of data loaded from memory is reused for many arithmetic operations, whereas spiking network simulations with simple neuron models typically involve lower arithmetic intensity and more irregular memory-access patterns. Accordingly, mostly modest speed-ups in this case have been reported—on the order of 1.5 to 3^24,27,28^—by comparing NEST GPU and GeNN^83,84^ to normal NEST. Together with the wide availability of multi-CPU systems, this presents massively parallel CPU-driven simulations of spiking networks as a competitive and cost-effective approach. Nevertheless, these performance results were obtained for single compartment models. Likely, the GPU speed-up with multi-compartment models will increase with increasing compartment numbers and model complexity^85,86^. For this reason, the NEST initiative intends to also provide code generation for the multi-compartment models in NEST GPU, as is already being developed for point neuron models.

Another interesting avenue for implementing networks of morphological neuron models is to leverage recent machine learning libraries with auto-differentiation and compilation capabilities, such as JAX^87^. In this way, Jaxley^88^ enables gradient-optimizable simulations of multi-compartment neuron models and networks. In the authors’ benchmarks, Jaxley achieved CPU runtimes comparable to NEURON. Although we do not provide a direct comparison here, extrapolating these results suggests that specialized compartmental code-generation pipelines optimized for cache locality, compartment vectorization, reduced gather/scatter operations, and event-based communication can outperform more general machine-learning-oriented execution frameworks in terms of pure simulation runtime. Although we do not assess GPU performance here, there is reason to expect that these same considerations will remain important there as well. It is therefore valuable to explore whether such neuroscience-optimized simulation pipelines can be combined with auto-differentiation frameworks in future work.

Converging evidence^35–37^ links the cellular and architectural organization of the cortex and hippocampus to neural dynamics across wake and sleep. At the cellular level, the distinct morphology of layer 5 cortical pyramidal neurons, with separate apical tuft and peri-somatic zones, enables compartment-specific integration of inputs. On a larger scale, inter-areal projections preferentially target the apical tufts, conveying contextual or internal signals, while sensory inputs predominantly impinge on the (peri-)somatic region. This organization points to apical amplification^89–91^, where during wakefulness selected neurons are boosted into high-frequency bursting in response to coincidences between top-down priors and bottom-up input. In turn, this amplification supports the formation of sparse, experience-specific assemblies at a faster rate than would be possible in point neurons. In contrast, during deep sleep, apical isolation is thought to decouple these dendritic compartments, reducing integration and enabling the optimization of local circuits^92^. The efficacy of learning algorithms based on apical mechanisms has been demonstrated in networks either by using abstractions of multi-compartment neurons^62,93,94^, or by emulations in models composed of single-compartment neurons^95,96^. These approaches, however, do not generalize to large-scale spiking networks due to the missing calibration of contextual and perceptual currents at growing scales, which is enabled by true simulation of apical mechanisms. We remark that the demonstration obtained through the *WTA-States* toy-model is not meant to describe a biologically accurate network. Rather, its purpose is to demonstrate the feasibility of leveraging apical dynamics for flexible and incremental learning in large-scale spiking networks^94,97^.

Recent years have seen a surge in the capability of artificial intelligence systems^6–8^. This can largely be attributed to an increase in scale^4^, both in terms of network size and compute, as the base network architectures have not fundamentally changed^5^. To elucidate their role in shaping high-level computations, dendritic trees will similarly have to be embedded in large-scale simulations implementing learning and memory. Only then can their computational role at the network level emerge. The work presented here implements the simulation technology necessary for this endeavor by proposing a flexible framework that enables the simulation of large-scale networks with dendrites in a massively parallel fashion. While the current implementation focuses on CPU systems with NEST, the use of the NESTML framework will enable code generation for GPUs as well as for a variety of emerging neuromorphic computing systems^98,99^.

## Methods

### Numerical integration of the compartmental dynamics

The membrane voltage in a multi-compartment neuron model, i.e., a model that follows (1), is integrated using the field-standard implicit Euler approach^9,100,101^, which ensures that the integration is stable. To that purpose, Eq. (1) is discretized in the following way:

2 $$\dfrac{{v}^{i}(t+h)-{v}^{i}(t)}{h}={F}^{i}\,\left[{{\bf{v}}}(t+h)\right],$$

where *h* is the integration step and *F*^*i*^ the entire right-hand side of Eq. (1) divided by the capacitance *c*^*i*^. Here, the unknown voltage ${{\bf{v}}}(t+h)=\left({v}^{1}(t+h),\ldots ,{v}^{N}(t+h)\right)$ at time *t* + *h* is featured inside the complicated non-linear function ${{\bf{F}}}\,\left[{{\bf{v}}}\right]=\left({F}^{1}\,\left[{{\bf{v}}}\right],\ldots ,{F}^{N}\,\left[{{\bf{v}}}\right]\right)$. We linearize this function around the known voltage **v**(*t*),

3 $${{\bf{F}}}\,\left[{{\bf{v}}}(t+h)\right]\approx {{\bf{F}}}\,\left[{{\bf{v}}}(t)\right]+{J}_{{{\bf{F}}}}\,\left[{{\bf{v}}}(t)\right]\cdot \left({{\bf{v}}}(t+h)-{{\bf{v}}}(t)\right),$$

where *J*_**F**_ is the Jacobian of **F**. By substituting (3) in (2), it follows that solving the matrix equation:

4 $$\left({{\bf{1}}}-h\,{J}_{{{\bf{F}}}}\,\left[{{\bf{v}}}(t)\right]\right)\cdot {{\bf{v}}}(t+h)=h\,{{\bf{F}}}\,\left[{{\bf{v}}}(t)\right]+\left[{{\bf{1}}}-h\,{J}_{{{\bf{F}}}}\,\left[{{\bf{v}}}(t)\right]\right]\cdot {{\bf{v}}}(t)$$

yields the next timestep’s voltage **v**(*t* + *h*). To efficiently solve this equation, the generated C++ code leverages the Hines algorithm^31^ (Fig. 1B, middle) with a level-parallel implementation. For the state variables of ionic channels and receptor currents, we employ the leap-frog scheme: state variables are computed first, advanced from $t-\frac{h}{2}$ to $t+\frac{h}{2}$ given the known voltage **v**(*t*). Then, spike-triggered updates are performed, which thus happen at $t+\frac{h}{2}$. Finally, the voltage is computed at *t* + *h* by solving (4), where we substitute the state variable values at $t+\frac{h}{2}$ in ${{\bf{F}}}\,\left[{{\bf{v}}}(t)\right]$ and ${J}_{{{\bf{F}}}}\,\left[{{\bf{v}}}(t)\right]$. The standard NESTML pipeline, featuring the ODE-toolbox^29^, is used to determine the best method of advancing the state variables. In many cases, the state variable evolution equations are *conditionally linear*; they are linear once the voltage and shared state variables such as ionic concentrations are known and assumed to be constant over the integration timestep from $t+\frac{h}{2}$ to $t+\frac{3h}{2}$. I.e. for a state variable equation of the form:

5 $$\dot{y}(t)=\dfrac{{y}_{\infty }\,\left[v(t)\right]-y(t)}{{\tau }_{y}\,\left[v(t)\right]},$$

the value at timestep $t+\frac{3h}{2}$ is computed as:

6 $$y\left(t+\dfrac{3h}{2}\right)={e}^{-\dfrac{h}{{\tau }_{y}\left[v(t+h)\right]}}\,y\,\left(t+\dfrac{h}{2}\right)+\left(1-{e}^{-\dfrac{h}{{\tau }_{y}\left[v(t+h)\right]}}\right)\,{y}_{\infty }\,\left[v(t+h)\right].$$

The resulting analytical integration to the next time-step (e.g., typical for Hodgkin-Huxley type channels) using propagators ${e}^{-\frac{h}{{\tau }_{y}\left[v(t+h)\right]}}$ is known as exponential Euler^102^. In more complex cases, the NESTML code generator falls back to the 5th order Runge-Kutta scheme^101^. This fallback path still treats voltage and shared state variables such as concentrations like constants over the integration time-step.

It should be noted that the integration engines use fixed time-step integration. Although this can yield inaccuracies for rapidly changing waveforms (such as AP shapes), the alternative of using adaptive time-step integration would be highly detrimental in distributed network simulations. In such use cases, any one neuron that is currently spiking would delay the entire network simulation. For this reason, network-oriented simulators like ours and CoreNEURON^10^ use fixed time-step integration exclusively.

### Code generation pathway

The NESTML code generation pipeline follows the standard NESTML pattern^30^ into which we inject our additional processing at specific steps. This general pattern starts with the initialization, where the correct code generator and other settings are configured based on user input. After that, the model is parsed, and syntactic and semantic checks are performed. Then the actual code generator is invoked, which inherits from a base NESTML code generator class.

#### Model parsing

The purpose of parsing the model is to verify the code’s syntactic correctness and to translate the NESTML code into an Abstract Syntax Tree (AST), a data structure that can be easily manipulated and traversed. The AST organizes all instances of syntactic elements that appear in the code into a hierarchical structure, and comes with an ASTVisitor class that, when called upon, traverses the tree recursively from any given node, using the “visitor” design pattern. For each ASTNode type, one can define visitor functions that can modify node content and extract information.

#### Mechanism-specific information collection

In the normal NESTML code generation context conditions checks (CoCos) verify that the NESTML code is semantically valid after building the AST. However, the compartmental case requires further information collection, as we need to distinguish between the various types of mechanisms. The original AST structure is not helpful to store this information, because it does not organize elements by their mechanism ownership. Since certain semantic checks can only be done when the ownership of elements is revealed, we inject our information collection into the CoCo process.

Each type of mechanism has its own information collector, which inherits from a base collector. From the inline that defines a mechanism—i.e., the ones marked with the @mechanism::{type} tag (cf. SI and Fig. S4)—we recursively search all referenced variables, their initialization expressions, functions, equations, and kernels. From the NESTML update block and our special case of the onReceive() function for self-spiking, we extract the parts associated with the mechanism. Finally, we utilize the ODE-toolbox to find solutions for the ODEs referenced per mechanism. This general collection process is applied to each mechanism. For the channel and concentration mechanisms, we furthermore check whether there are parameter combinations that result in inputs that are identically zero, so that we can omit their simulation from those compartments. This is a key aspect of the modularity of the compartmental feature.

The synaptic processing performs very similar information collection as is the case for neurons in terms of the structure of collected elements, states, functions, ODEs, and so on. We don’t insert this into the CoCos but instead into the original synapse processing because we don’t perform additional semantic checks that rely on a mechanism-wise distinction of elements, as is the case for neurons. In addition, we collect the update block, the spiking ports, determine which is the pre- and post-synaptic port based on the code generator options, and collect the onReceive() blocks for those ports. We collect variables that potentially refer to a postsynaptic neuron variable. As for the neurons, we utilize part of the original transformations for inlines and convolutions.

#### Converting equations to C++ expressions

After the CoCos have been performed successfully, the code generator is invoked for each mechanism that has been parsed. Here, we start by invoking the parts of the standard neuron processing pipeline where the incoming spike buffers for synapses are generated, inline references are replaced with their expressions, and convolution calls are replaced by a new variable that will contain their result.

We insert an additional step into the template namespace setup. The namespace is a dictionary that contains all the data and Python functions referenced inside the template. In this additional step, we perform modifications to AST nodes in our collected mechanism dictionaries, which we could not perform during the original collection, as it requires the invocation of the NESTML parser, which in turn invokes the CoCos, leading to a circular dependency.

During this information enrichment process, we parse the resulting expressions from ODE-toolbox, add the propagators to the internals block, and collect the updated expressions in which the inlines and convolutions have been substituted. We need to parse our new expressions and all new resulting variables as AST nodes because the already defined C++ printers in NESTML rely on this input format.

### The C++ code structure

We generate the C++ code with Jinja (https://jinja.palletsprojects.com/en/stable/), a templating language that can process standard Python data structures and to which one can pass Python objects that can be used in the Jinja logic blocks. Jinja also provides control structures with a Python-like syntax.

#### The generated C++ files

A module, as defined by the user, may include an arbitrary number of models that can be passed to the generator. The class that registers the module is an extension of the NESTExtensionInterface class in which the contained models are registered with the simulator. The name of this generated file is <module_name>_module.cpp.

The top model classes are defined in <model_name>_nestml.cpp/.h These inherit from the NEST ArchivingNode class. When writing a custom NEST model in C++, one must define certain functions in the model class like update(), pre_run_hook(), and set_status(). The update() function is called at every timestep during the simulation. The pre_run_hook() function may be used to internally configure the neuron properly before the simulation starts. The set_status() function may be called explicitly from the Python interface or is called implicitly when assigning a member variable of the Python neuron object. It receives a dictionary with entries corresponding to the user-defined or certain predefined states and parameters of the neuron. In our case, we also use this function to add new compartments and receptors to the compartmental tree by detecting an entry with id “compartments” or “receptors”, which internally is a dictionary in which we expect to find the parent node id and a corresponding parameter dictionary.

In the cm_tree_<model_name>_nestml.cpp/.h files, the compartmental tree data structure is defined. This class represents the user-defined tree structure of the model through CompartmentNode attributes that contain all parameters and states that are part of the passive dynamics, which are implicitly included in all compartmental models. The tree class, as container, owns the root compartment and has a list of pointers to all compartments in the order in which they were added. This class integrates the passive dynamics directly and triggers the integration of compartmental dynamics not through the compartment class but through the NeuronCurrents class. Finally, it combines the integrated compartment internal dynamics and performs the integration of cable dynamics through the Hines algorithm.

The NeuronCurrents class is defined in the cm_neuroncurrents_<model_name>_nestml.h file. It primarily contains an update function that combines integration results from all mechanisms across the neuron and manages the instances of mechanisms that the user adds to the compartments of the neuron. The cm_neuroncurrents_<model_name>_nestml.h and .cpp also define the mechanism classes, which are generated based on the mechanisms that have been defined by the user. Each mechanism class defines three functions that are relevant to the core functionality. The f_numstep() function, which is called at every update of the simulator, integrates the defined dynamics. Synaptic dynamics are added to the f_numstep() in case of the receptor-synapse mechanisms. The pre_run_hook() function precomputes quantities that are not dependent on any evolving state. And a f_self_spike() function, which is the reduced form of the OnReceive(self_spike) function in the model, which is always called when the neuron spikes. In all mechanism types, except for the concentration type, the f_numstep function returns the current induced by the mechanism.

#### Micro-parallelization through vectorized C++ code

One of our goals was to achieve a further level of parallelization—beyond the MPI OpenMP parallelization that NEST implements at the network level—by vectorizing the integration of mechanism dynamics. Vectorization is a parallelization technique that relies on a so-called vector register on the CPU, which can contain multiple values of the same type on which the same operation is performed in one step. To make this possible, the data on which computation is performed needs to be provided in the form of an array or array container, such as the vector datatype in C++. We vectorize all instances of the same mechanism type added to the whole neuron. Each object of a mechanism class thus encapsulates all its instances for the entire neuron, except for those cases in which the parameters result in a contribution that is identically zero (up to machine precision). Each defined state and parameter is stored as a vector, where each entry contains the state or parameter value in a single compartment. When integrating the associated dynamics, we can loop through all instances. By marking this loop with #pragma omp simd, we tell the compiler to vectorize the operations contained in this loop. Additionally, we need to consider that we also call functions within this loop. These functions need to be vectorizable. We found that we need to do the following to get the compiler to consistently vectorize our functions. When declaring these functions we need to add the pragma #pragma omp declare simd and mark them as inline with __attribute__((always_inline)) in addition to the inline keyword. Furthermore, we need to compile with the flags -march=native and -ffast-math. We are aware that the -ffast-math flag may introduce numerical artefacts due to the nature of floating-point arithmetic and, depending on compiler and platform behavior, may also enable relaxed handling of subnormal values such as flush-to-zero semantics. In our benchmarks, however, we did not observe numerical issues attributable to these effects.

### Simulation details

#### Benchmarking and scaling experiments

All benchmarking and scaling experiments were performed on the Jureca supercomputer at Forschungszentrum Jülich^51^, except for the profiling experiments, where we used a laptop with an Intel(R) Core(TM) i5-4210H CPU @ 2.90GHz and 16 GB DDR3 1600 MHz RAM. We used NEST version 3.9 and NEAT version 1.0. In the comparisons with NEURON, we used version 9.0.1, and in the comparison with Brian2, we used version 2.10.1.

The artificial morphologies have a soma with radius 10 μm. The star neuron, for *N* compartments, was then equipped with *N*−1 cylindrical branches of length 10 μm and radius 1 μm, which each were converted into a single electrical compartment using standard geometrical principles^9^. The linear neuron had a single dendritic branch of radius 1 μm and length 1000 μm, which was discretized into *N*−1 compartments. Na^103^ resp. K^104^ currents were distributed at the soma with densities 1.71 × 10^6^ μS/cm^2^ resp. 0.766 × 10^6^ μS/cm^2^, and with 1/10’th of the somatic density in the dendrites. Finally, passive leak conductance and reversal were fitted to obtain a membrane time-scale of 10 ms at an equilibrium potential of −75 mV.

Synaptic conductances in most simulations followed the dual exponential profile^105^:

7 $${i}_{{{\rm{syn}}}}=g\,\left({e}_{r}-v\right),\,{\mbox{with}}\,g=w\,n\,\left[{\tau }_{r},{\tau }_{d}\right]\,\left({e}^{-t/{\tau }_{d}}-{e}^{-t/{\tau }_{r}}\right).$$

with parameters *τ*_*r*_ = 0.2 ms, *τ*_*d*_ = 3 ms, and *e*_*r*_ = 0 mV they represented excitatory (exc) AMPA synapses, whereas with parameters *τ*_*r*_ = 0.2 ms, *τ*_*d*_ = 5 ms, and *e*_*r*_ = − 80 mV they represented inhibitory (inh) GABA synapses. $n\,\left[{\tau }_{r},{\tau }_{d}\right]$ is a normalization factor so that the maximum of the conductance windows is the synaptic weight *w*.

In the single-neuron benchmarks, we only used AMPA synapses, one in each compartment, and adjusted the input Poisson spike rate such that the total input rate to the neuron was 200 Hz. Excitatory weight *w* was 4 nS. In the network benchmarks, we also used inhibitory neurons and GABA synapses, and had five connection weights: *w*_exc→exc_ = 1 nS, *w*_exc→inh_ = 2 nS, *w*_inh→exc_ = 8 nS, *w*_inh→inh_ = 2 nS, and *w*_ext→exc_ = 2 nS. The neurons in the network were connected randomly with a fixed in-degree. The exc → exc connection had a total in-degree of 200, and the exc → inh, inh → exc, and inh → inh connections had an in-degree of 400. These incoming connections were distributed homogeneously over all compartments. Finally, the Poisson input generators—with firing rates of 400 Hz—were connected in a one-to-one fashion to the somata of the excitatory neurons. To ensure a fair comparison, we tested the optimal layout in terms of MPI processes/OpenMP threads for each simulator independently (Fig. S3).

#### Ca-AdEx simulations

The Ca-AdEx model featured the dynamics shown in Fig. 5B, with parameters adapted from Pastorelli et al.^16^. Additionally, appropriate input receptors were added for each simulation paradigm. Specifically, for the BAC firing protocol, we had a somatic input current receptor port that received a short current pulse of 5 ms duration and 1000 pA amplitude, and a dendritic current-based synapse with a dual exponential profile (*τ*_*r*_ = 0.2 ms, *τ*_*d*_ = 10 ms) of weight 400 pA. To simulate the STDP protocol, we used an AMPA synapse with an initial weight of 10 nS and a maximal weight of 20 nS. Other STDP parameters can be consulted in the code (see also Fig. S7).

#### Winner-take-all network

The network described as a use-case for the NESTML compartmental modeling framework represents a specific customization of the more general *WTA-States* toy-model(https://github.com/APE-group/WTA-states). *WTA-States* is used for the initial calibration of the cortical layer in a more complete thalamo-cortical plastic spiking model capable of incremental learning and sleep cycles (*ThaCo*^95^,^96^) through the emulation of apical mechanisms. The ThaCo model is currently under extension to leverage the *Ca-AdEx* two-compartment neural model^16^. In *WTA-States*, based on a Winner-Take-All (WTA) mechanism, populations of recurrently connected excitatory neurons, the neural assemblies, are able to learn memories through the combination of a specific contextual signal with a sensory input. The WTA mechanism is obtained through competition among populations of excitatory neurons connected towards the same population of inhibitory neurons.

The model, implemented in NEST, can be configured to work with both two-compartment (Ca-AdEx) and point-like (AdEx) excitatory neuron models, showing how the performance takes advantage from the apical mechanism in the Ca-AdEx model. Several parameters of the network, such as the number of neurons in each population and the number of populations, can be defined by the user through configuration files in YAML format. In this work, the WTA-States network was configured with four excitatory populations, each one composed of 20 recurrently connected neurons. A single inhibitory population of 20 neurons is connected to all the excitatory populations, implementing the WTA dynamics. The connectivity, both recurrent intra-population and between excitatory and inhibitory neurons, is implemented using *all-to-all* connections with the following synaptic weights: *w*_exc→exc_ = 12 nS, *w*_exc→inh_ = 7.0 nS, *w*_inh→exc_ = 1.5 nS, *w*_inh→inh_ = 1.0 nS, where the synaptic receptor currents follow the dual exponential profile (7) with *τ*_*r*_ = 0.173 ms, *τ*_*d*_ = 0.227 ms, and *e*_*r*_ = 0 mV for the excitatory synapses and *τ*_*r*_ = 1.73 ms, *τ*_*d*_ = 2.27 ms, and *e*_*r*_ = − 85 mV for the inhibitory synapses. In addition, excitatory populations are stimulated with Poisson noise: each neuron receives input from 20 Poisson generators firing at 600 Hz, through a synaptic weight *w*_*noise*_ = 1.0 nS. Note that the passive electrical parameters of the point neuron were tuned to have the same input resistance as the two-compartment neuron. However, the two-compartment model had additional membrane leak not incorporated in this analysis because of the Ca^2+^-activated K-current, resulting in slightly lower overall network firing rates for identical weights. To correct this, we increased *w*_exc→exc_, *w*_inh→exc_ and the external input weight with a scale factor of 1.1 in the two-compartment case, which resulted in approximately equal firing rates for the corresponding populations.

New memories were written into the network using a contextual signal in the form of a Poisson stimulus of 3000 Hz during a 25 ms window, injected through dual exponential current-based synapses with *τ*_*r*_ = 0.173 ms, *τ*_*d*_ = 10 ms, and synaptic weight *w*_*stimulus*_ = 4.0 nA, into selected excitatory populations. This excitatory stimulus was preceded by 200 ms of inhibitory Poisson input at a rate of 200 Hz, through conductance-based synapses with *τ*_*r*_ = 1.73 ms, *τ*_*d*_ = 2.27 ms, *e*_*r*_ = − 85 mV, and weight 2 nS. Recurrent connectivity in the targeted populations was made plastic through a multiplicative STDP model^106^ with *λ* = 0.1, *α* = 1.05, *τ*_+_ = 20 ms.

All connections impinge on the peri-somatic compartment, except for the contextual signal that, only when using two-compartment Ca-AdEx neurons, targets the distal compartment, allowing for the activation of the apical amplification mechanism. Note that the contextual stimuli were identical in the two-compartment and point-neuron networks.

## Supplementary information

Transparent Peer Review file

Supplementary Information

## Data Availability

Benchmarking results are available at https://jugit.fz-juelich.de/w.wybo/ca-adex/-/tree/paper-revision/data or in the Zenodo archive https://doi.org/10.5281/zenodo.22878144.

## Code availability

Code to reproduce the simulations in this paper is available at https://github.com/WillemWybo/WTA-states/releases/tag/v1.0 for the winner-take-all network and at https://jugit.fz-juelich.de/w.wybo/ca-adex/-/tree/paper-revision for all other simulations. A Zenodo archive is available at https://doi.org/10.5281/zenodo.22878144.
