# Supplementary material for: A NEST-based framework unlocks massively parallel simulation of networks of multi-compartment neurons with customizable subcellular dynamics
Source: Commun Biol. 2026 Oct 27;9:1411. doi: 10.1038/s42003-026-11157-0 (PMC13714284; doi:10.1038/s42003-026-11157-0)
Supplement: Supplementary file 1 [file 42003_2026_11157_MOESM1_ESM.pdf]

nature portfolio

Peer Review File

A NEST-based framework unlocks massively parallel simulation of networks of multicompartment neurons with customizable subcellular dynamics.

Corresponding Author: Dr Willem Wybo

This file contains all reviewer reports in order by version, followed by all author rebuttals in order by version.

Version 0:

Reviewer comments:

Reviewer #1

(Remarks to the Author)
Overall

This work represents a solid technical achievement that addresses a genuine computational need in neuroscience, but suffers from some gaps in positioning and evaluation gaps that undermine its impact.

The technical implementation is well-executed. The NESTML-to-C++ compilation pipeline is elegant, the vectorization approach is efficient, and the integration with NEST's parallel infrastructure leverages existing optimizations effectively. The work is biologically well-motivated, as dendritic calcium dynamics and apical amplification are critical to neural information processing. The Ca-AdEx model example demonstrates meaningful differences between compartmental and point-neuron learning.

The software architecture appears robust and the scaling performance within the framework is impressive.

The manuscript's major weakness is the somewhat outdated competitive ecosystem. By comparing only against NEURON while ignoring
e.g. PyTorch-based spiking frameworks such as snnTorch, Norse, SpykeTorch and differentiable ODE approaches, the manuscript positions this work against 2010-era alternatives rather than contemporary solutions.

The NEURON comparison, while showing real performance gains, tests only simple scenarios that may not reflect realistic model complexity where NEURON's flexibility become relevant. Comparisons were not performed against CoreNEURON, which is much more optimized than standard NEURON. The lack of complex synaptic models in the benchmarks is particularly puzzling.

The validation scope is too narrow. One biological example, however compelling, doesn't demonstrate the framework's breadth or establish when researchers should choose the NEST/NESTML approach over alternatives.

The biological insights about dendritic learning are valuable and could stand more prominently as a contribution alongside the technical implementation.

I suggest the following modifications to the manuscript and related computational experiments:

- Acknowledge modern alternatives and position this work as advancing compartmental modeling specifically within NEST, not solving a
general field-wide gap
- Include at least one comparison against a spiking neuron implementation based on auto-differentiation framework

- Demonstrate framework capabilities beyond calcium dynamics with additional biological examples

- Include complex synaptic models and CoreNEURON comparisons or acknowledge the limitations of current benchmarks

- Clearly state when researchers should choose the NEST/NESTML approach versus alternatives

Following are my detailed comments per section:

1. Introduction

- Clearly articulates the computational bottleneck between detailed single-neuron simulators (NEURON) and large-scale point-neuron
networks (NEST)

- The approach of leveraging NESTML within NEST's parallel framework is well-motivated and appears novel

- Reasonable positioning between existing extremes -- more detailed than point neurons but more scalable than full biophysical models;
however, a number of solutions for this already exist, such as the Brian simulator.

- Could better quantify the performance limitations of existing approaches

- More expanded comparison of computational trade-offs would strengthen the gap argument

2. Methods

While using implicit Euler for the membrane voltage equation is well-justified for stiff systems typical in biophysical neuronal modeling, and the use of the Hines algorithm leads to really efficient compartmental tree solving, there are some concerning issues with the numerical integration method as described.

First, the scheme makes several approximations that interact problematically:

- Voltage linearization (eq. 3): F is linearized around v(t)
- Leap-frog assumption: State variables see voltage as constant during [t, t+h]

- "Exponential Euler exactness": The claim of "analytical integration" is misleading -- exponential Euler is a semi-analytical method that is only exact given the constant-voltage assumption. But during rapid spiking, voltage can change by ~100 mV in 1-2 ms, severely violating this assumption.

There are temporal inconsistencies in the order of operations:

- Voltage uses implicit Euler at discrete times: v(t), v(t+h)
- State variables are evaluated at staggered half-steps: y(t+h/2)
- The "analytical" integration of state variables assumes
piecewise-constant voltage, which creates a half-step phase lag
between voltage and conductances

During fast events (spike upstroke), this staggered timing could produce delayed channel activation relative to voltage, and therefore
a broadening of action potentials and incorrect recovery dynamics purely as a result of numerical integration artifacts.

Furthermore, equation (3) linearizes F around v(t), and equation (4) appears to solve once. Is there Newton iteration to refine the
solution? For strongly non-linear channels, such as sodium, single-step linearization will be very inaccurate, and the inaccuracies will compound at each spike. How is the Jacobian J_{F} computed?

The use of mixed-order methods is not really justified or validated. Voltage is solved with 1st order implicit Euler, state variables are solved with "exponential Euler" (2nd order if voltage truly constant), or RK5 (5th order for complex cases). The overall error is thus dominated by the 1st-order voltage integration, which means the extra steps of RK5 are somewhat wasteful. More importantly, users may be misled by "5th order Runge-Kutta" into thinking they have high accuracy when the coupling structure limits the overall accuracy.

Recommendations

- Clarify "exponential Euler": explain it's exact only under constant-voltage assumption

- Add validation section: Test different timesteps, show convergence tests and comparisons with high-accuracy reference solutions

- Discuss limitations: be explicit about when this scheme may introduce non-negligible errors (for instance when simulating

interneurons with high firing rates)

- Error estimation: Provide tools or guidelines for users to assess numerical accuracy

3. Results

- The NESTML to C++ compilation pathway is well-explained and technically sound; 8 compartments per CPU through vectorization shows good optimization

- Strong scaling results (Fig 2F) demonstrate good parallel efficiency

- Ca-AdEx combining abstract and detailed dynamics is innovative

However there are some critical gaps:

- Comparing only to NEURON ignores CoreNEURON and modern PyTorch-based and JAX-based frameworks (snnTorch, Norse, SpikingJelly, Rockpool, etc.)

- There is no comparison to GPU-accelerated spiking frameworks

- The only somewhat biophysical example is Ca-AdEx model with BAC firing

- Single-shot learning is interesting but doesn't validate framework breadth

- There is no comparison across different neuron types, channel types, or network architectures. One example simply doesn't establish broad utility.

- The performance claims are ultimately unclear: without modern baselines, it unclear if this enables previously impossible simulations / research questions.

The manuscript should at least be able to answer the following questions:

- How does the NEST/NESTML framework perform vs. the PyTorch spiking simulation ecosystem or the Jones & Kording-style ODE approaches in JAX?

- How does the NEST/NESTML framework perform with GPU acceleration?

- There were limited morphologies tested: only star and linear dendrites. How do real morphologies behave?

- Some other differentiable biophysical modeling approaches to consider and compare against:

- Lohoff et al. "SNNAX -- Spiking Neural Networks in JAX" (arXiv:2409.02842, 2024)
- Heckel & Nowotny "Spyx: A Library for Just-In-Time Compiled Optimization of Spiking Neural Networks" (arXiv:2402.18994, 2024)
- Confavreux et al. "Meta-learning biologically plausible plasticity rules with random feedback pathways" Nat. Comm. (2023)

- Very limited single neuron benchmarks (Fig 2A-D):
- Na+ and K+ channels (Hodgkin-Huxley type)
- No synaptic models: these are purely intrinsic membrane dynamics
- NEURON vs NEST: Fair comparison for channel kinetics only

- Limited network benchmarks (Fig 2E-F):
- Synapses present but unspecified? Networks have connections but no details on synaptic model complexity.
- No NEURON comparison: Only NEST scaling, not NEST vs NEURON for network synapses
- Very basic connectivity: Fixed in-degree, random connections

- Synaptic Model Validation (Fig 3D):
- NEST vs NEST only: Compares standard NEST STDP with their NESTML implementation
- No NEURON involvement: There isn't a NEURON comparison at all

A fair comparison would need:

1. Complex synaptic models: AMPA, NMDA, GABA with realistic kinetics
2. Multiple receptor types: Testing computational overhead of diverse synaptic mechanisms
3. Plasticity rules: Comparing equivalent plasticity implementations across both simulators

4. Realistic connectivity: Testing performance with biologically realistic synaptic densities

Recommendations

- Expand competitive comparisons vs. CoreNEURON, PyTorch-based, JAX-based, other differentiable approaches

- Include GPU-accelerated framework comparisons

- Clearly answer: when should researchers choose NEST/NESTML over alternatives?

- Test diverse receptor kinetics: AMPA, NMDA, GABA with realistic multi-state models, short-term plasticity, fair NEURON comparison
with long-term plasticity

- Beyond the single Ca-AdEx example, demonstrate or discuss multiple neuron types, fast-spiking, adapting, etc.

- Test or at least discuss diverse channel combinations, realistic morphologies, various connectivity patterns, synaptic densities

- Test performance across a range of firing regimes, e.g. sparse vs. high-frequency activity

- Address numerical accuracy for different regimes: e.g. timestep requirements for regular-spiking, fast-spiking neurons, different
morphological complexities

Without these additions, readers cannot assess whether this work represents a meaningful advance over existing solutions or enables previously intractable research questions.

Reviewer #2

(Remarks to the Author)
Thank you for this interesting, timely and well-written paper. Frameworks for simulating large networks of 'few-compartmental' neurons are very much needed in the field of computational neuroscience and spiking ML. Furthermore, the vectorising of compartmental updates is both a good ideas and, I imagine, a pragmatic way to exploit extra parallelism without any changes deeper in NEST itself...

My only major criticism of this paper is that it really should compare against Dendrify which aims to solve the exact same problem but builds on the Brian2 simulator rather than NEST. From my understanding of Brian2 and Dendrify, I SUSPECT the equations it generates will be solved using a standard Brian2 solver rather than the Hines method so, it will scale less well with more compartments but, nonetheless, I think figure 2 B,C and D should be extended to include a comparison against Dendrify. I also BELIEVE Dendrify is compatible with Brian2CUDA for GPU simulation.

Other than that, just a few minor things:

* Line 36 - I think it would be good to be explicit that you are talking about attention here
* Line 73 - I assume this 8-way parallelism is for double-precision float on an AVX-512 CPU. It would be good to make this explicit.
* Line 85 - STDP
* Line 89 - There's a stray uppercase V in equation 1 which I think should be lower case. Also I think it would be good to use different parentheses for 'functions' e.g. i_c^i and actual bracketing e.g. around g^j - v^i in the passive system. Finally, I don't think you ever define S_r - presumably it's a weighted sum of incoming spikes
* Line 104 - would be nice to link back to the equation here and repeat that active currents are i_c^i and i_r^i.
* Line 105 - "allowing modern CPUs TO integrate"
* Line 140-152 - How come this model is constructed to fire at such a low firing rate (1-2spike/second)? That is definitely going to help with being "distributed over many CPUs with little overhead"
* Line 173 - I assume this is referring to e-prop. It would be good to cite Bellec et al. here.
* Figure 3 - I think the font used for the code is WAY too small here. Maybe re-arrange the panels so the code flows across two columns or something. Also, in panel C, how come the dendritic and somatic stimulus currents are different shapes?
* Line 206 - Where does this ten frame per/second number come from?
* Line 230 - The reason GPUs are particularly good at matrix multiplication and not so great at SNN simulation is that matrix multiplication has a very high arithmetic intensity i.e. each matrix tile read from memory gets a lot of maths done to it which is not really the case for SNNs with simple neurons
* Line 236 - 10.1038/srep18854 would be good to cite here as they already explored this a little, finding that "Izhikevich neurons are much simpler than Hodgkin-Huxley neurons, and therefore can be simulated faster both on GPUs and on the CPU, but the relative speedup of the GPU compared to the CPU was ten folds higher for the benchmark which uses Hodgkin-Huxley neurons"

* Line 568 - an additional (and more insidious) issue with fast math is that the flush to zero rounding mode it sets tends to be per-thread so messes up other stuff as well. I don't know if that's an issue with NEST as maybe all the fast math code only runs in a worker thread.

Reviewer #3

(Remarks to the Author)
This manuscript describes a new framework for constructing and simulating neural network models based on neuronal models that are intermediate in complexity and biological realism between point neuron models and morphologically detailed model neurons. One expected major area of application is modeling the effects of nonlinear dendritic processes and related plasticity phenomena on network dynamics and learning. This is demonstrated directly in the paper by providing a new network model of the modulation of learning by top-down inputs to the distal dendrites of layer 5 neocortical pyramidal neurons.

This works builds heavily on previously developed software infrastructure related to the NEST simulator, but extends existing tools such as the NESTML model description language in clever ways to enable the efficient simulation of morphologically simplified multi-compartmental models with arbitrary biophysical mechanisms and synaptic plasticity. The technical aspects of the implementation are described in sufficient detail to enable the understanding of the underlying concepts, and links to the source code are also provided. A limited set of benchmarks is also included and shows good scaling of the implementation to more neurons, compartments, and CPUs.

My main concern related to the software framework described is that it is not clear to me how easy it would be for an average user to implement their own models based on the available information. The models showcased by this paper were built with the participation of the researchers who are also actively involved in the development of the underlying tools such as NEST and NESTML, and building the models seemed to require not just deep knowledge of the technical details of these tools, but also their further development. This raises at least two questions. First, can users now expect that the latest versions of the necessary software tools will support a wide range of modeling scenarios without requiring further changes to the codebase? And second, is the documentation of the model-building process and the underlying tools themselves sufficiently detailed to enable new users to apply this framework in their own research? In general, is the framework sufficiently mature to support uninitiated users, or is it currently more suitable for those who are already familiar with the technical details of the underlying tools?

The last part of the Results section presents a network model in which an appropriately chosen pattern of inputs to the dendritic compartments of excitatory neurons leads to the rapid strengthening of connections between these cells. This simulation is clearly a nice demonstration of the capabilities of the modeling framework described in the first part of the paper; however, it is not entirely clear whether the authors also consider these results as a novel proposal regarding the actual mechanisms that mediate the effects of top-down inputs on learning in the neocortex. If this is the intent of the authors, then I think that this part should be significantly extended, and more motivation, analysis and discussion should be provided. For instance, the choice of parameters in the winner-take-all network should be further motivated, especially since some parameter values (including some specific synaptic conductances and time constants) appear to be outside the range that is generally considered to be biologically plausible. In addition, claims about coordinated vs. random firing should be properly supported by appropriate synchrony measures and statistical analyses. Furthermore, claims such as the one that, without dendritic amplification, one-shot learning would require contextual inputs of “unrealistic strength” should also be properly supported by quantitative data and comparison with relevant experimental results. On the other hand, if the sole purpose of this section is to show the applicability of the software framework to this type of question, then this should be explicitly acknowledged, but it may not be necessary to provide all the detailed analysis that I referred to above – although some further discussion of the assumptions and findings in the context of the relevant experimental literature would still be welcome.

I also have some more specific comments and questions about the manuscript, which are listed below.
1. In the context of the usability of the software by users with varying levels of expertise, some discussion of error handling and error messages in the different components of the software could be useful.
2. It would be useful to include some discussion of the limitations of the current software framework, and whether there are any plans to extend its capabilities.
3. A technical detail that I think is important from the perspective of efficiency: Does NEST take into account the complexity of neuronal models when the cells are distributed across CPUs?
4. The examples in the repositories appear to require the NEAT tool, but it is not clear from the manuscript why it is necessary and how it is used in this case.
5. How will the long-term availability and support of both the software tools and the example models be ensured?
6. There are several grammatical and spelling errors that should be corrected. For example, there is an unfinished sentence in line 687; “trough” appears instead of “through” multiple times on page 32; the leak reversal potential is listed as 75 mV (rather than -75 mV) in line 581; there are also typos in lines 186, 198, 205, 592, and 692 that should be easy to fix.

Version 1:

Reviewer comments:

Reviewer #1

(Remarks to the Author)
Overall

I thank the authors for diligently addressing my concerns. The majority of the issues I raised have been substantively addressed.

The expanded benchmarks against CoreNEURON, Brian 2, biophysically realistic Blue Brain Project cells, and network-level NEURON/CoreNEURON comparisons directly answer my concerns about the competitive ecosystem and validation breadth, and the clarification of the numerical scheme and use-case positioning is appropriate. I recommend minor revisions: the clarification items below are requests for more precise language and a few specific clarifications, and should not be considered objections to the core contribution.

Adequately addressed

- The CoreNEURON comparison is now included across single-neuron, realistic-cell, and network benchmarks (Figs 2-4), resolving my main competitive-baseline concern;
- The addition of Brian 2 SpatialNeuron as a baseline, with explicit justification for preferring it over Dendrify, is well handled;
- The new Fig. 3 with five biophysically realistic BBP models containing diverse Na/K/Ca channels and AMPA+NMDA+GABA receptors addresses both the "single biological example" and "diverse neuron/channel types" concerns;
- The network benchmark now includes NEURON/CoreNEURON comparisons rather than NEST-only scaling (Fig. 4);
- The use-case positioning (large-scale distributed CPU networks with biophysical learning) is now stated clearly in the introduction and discussion;
- The timestep-convergence study (Fig. 2G) and inclusion of fast-spiking interneurons appropriately address the requested accuracy-vs-regime analysis;
- The fixed-timestep justification on network-synchronization grounds is reasonable and well argued;
- The minor notation, citation, and clarity items (S_r definition, AVX-512 explicitness, e-prop citation, fast-math discussion, error handling, load-balancing limitation, NEAT usage) are satisfactorily resolved.

Requests for clarification

- The auto-differentiation comparison is declined on a transitive argument (Jaxley ~= NEURON, NEST beats NEURON); this is understandable given that Jaxley appears to be the only cited tool that supports multicompartment morphologies, but relies on cross-paper, cross-hardware numbers rather than a controlled measurement;

- Multi-state synaptic kinetics and short-term plasticity were not tested; the claim that multi-state schemes would not "induce disproportionate performance degradation" is an unsupported assertion and should be stated explicitly as untested rather than presumed negligible;

- Please clarify whether a single receptor mechanism can mix continuously integrated quantities (e.g., calcium, NMDA Mg block) with event-driven, spike-triggered updates of other quantities, analogous to NEURON's DERIVATIVE/BREAKPOINT/NET_RECEIVE structure;

- Relatedly, please specify the timing semantics of how a spike delivered via S_r interacts with the leap-frog staggered integration of continuous states within a step (i.e., whether event-triggered modifications are applied at the voltage time point or the staggered half-step), as this bears on accuracy for mechanisms coupling calcium, voltage, and spike timing.

On the biological framing of the network model (also related to Reviewer 3's comments)

- There is a contradiction in the revised manuscript between its framing and its demonstration: the single-cell benchmarks (Fig. 3) use genuinely biophysically realistic BBP-derived models, but the flagship network demonstration (Fig. 6) is a WTA "toy-model" that the authors now explicitly state is "not meant to describe a biologically accurate network."
- This creates an inconsistency: the abstract and discussion advance mechanistic biological conclusions (single-shot association learning, ~10 Hz memory formation), yet these conclusions rest on a network the authors disclaim as non-biological, with several parameters (e.g. 3000 Hz contextual Poisson, $w_{stimulus}$ = 4.0 nA) outside physiological ranges;

- A possible constructive way to resolve this contradiction: the NEURON/CoreNEURON test suites include several reduced models derived from realistic biophysical network models, at least one of which could serve as a biologically grounded network demonstration. This would simultaneously strengthen the realism of the flagship result and provide controlled network-level NEURON/CoreNEURON comparison, addressing both concerns at once and reusing the cross-simulator workflow the authors have already built;

- The requested synchrony measures and statistical support for the coordinated-vs-random firing claims were not added and would further strengthen whichever framing the authors choose.

On the numerical integration scheme in Methods

I appreciate the authors' clarifications regarding conditional linearity and the exponential-Euler / RK5 fallback, and the new timestep-convergence analysis in Fig. 2G is a valuable addition. The empirical agreement with NEURON across timesteps is reassuring. I'd like to push on one point, though, not to ask for more work for its own sake, but because I think the authors are unusually well-positioned to make a considerable contribution.

My original questions about Newton iteration and the Jacobian, and about the half-step phase lag between voltage and conductances, were largely answered by appeal to NEURON's "cnexp" scheme as the established standard. I note three things about this framing:

First, "matches the gold standard" is a useful sanity check, but as a primary validation strategy it tends to reproduce the reference implementation's behavior, including any of its approximation artifacts, as opposed to independently characterizing the error. If the computational neuroscience field has only one trusted reference, that reference effectively becomes a black box whose accuracy can't be cross-checked. The authors have built a genuinely independent implementation of compartmental integration. This makes them one of the few research groups in a position to provide an independent point of comparison rather than deferring to the first. A small analysis establishing the scheme's accuracy on its own terms (e.g., against a high-accuracy reference solution at very small timestep, which Fig. 2G partly does, and ideally an analytic or near-analytic case) would be more valuable to the community than NEURON-equivalence alone, and would strengthen the manuscript's standing as a reference tool.

Second, on the specific technical question: to my understanding NEURON's default solver does perform some form of iterative (Newton-type) refinement of the voltage solution rather than a single linearization, so the "cnexp" comparison may not fully address whether a single solve of eq. (4) is adequate for strongly nonlinear channels at the spike upstroke. Relatedly, stiffness issues arising from features such as dendritic spines are precisely the kind of regime where single-step linearization error can matter, and these deserve study by the community beyond what any single simulator's defaults provide. I'm not asking the authors to solve this in full, but only to either describe explicitly how eq. 4 is solved (single solve vs. iteration) and how J_F is computed, or to state plainly that single-step linearization is used and to bound the regime in which it is reliable.

Third, and I raise this constructively, because I think it actually helps the paper: if NEURON is positioned as the accuracy gold standard against which the method is validated, this is in contradiction with the manuscript's central motivation for offering an alternative. The paper's case for NEST/NESTML rests on efficiency and scalability, not on accuracy superiority, and that's entirely legitimate. But it would be worth making explicit that the contribution is a performance-and-scalability advance at _equivalent_ accuracy to the established reference, rather than letting "validated against NEURON" and "an improvement over NEURON" stand side by side without reconciliation. A sentence framing the accuracy claim as parity (within characterized bounds) and the contribution as scalability would resolve this cleanly.

None of this diminishes what is a substantial and carefully executed body of work; these are requests for sharper framing and a modest independent error characterization, both of which I think would raise the manuscript's value as a community resource.

Reviewer #2

(Remarks to the Author)
Thank you for your hard work on this revision - I think your changes have addressed all of my concerns. I can only imagine adding comparisons to two additional simulators was very time-consuming but, in this case, I really do think that it has significantly improved the paper.

Reviewer #3

(Remarks to the Author)
I thank the authors for carefully addressing my earlier comments and clarifying several issues. I have no further comments on the manuscript.

Version 2:

Reviewer comments:

Reviewer #1

(Remarks to the Author)

I thank the authors for their thorough and responsive revision, and in particular for the addition of the analytical Green's function validation (Fig. S2). Testing against an independent, closed-form reference significantly strengthens the standing of the NEST framework as a community reference tool.

On the synchrony/statistical-support request for the WTA network demonstration: given the substantial validation work that the authors have already undertaken, I agree that a fully biologically-constrained network model is more appropriate for a separate paper.

My concerns have been addressed. I have two remaining requests, both purely textual edits. With these in place, I have no further comments and do not require further review.

1. Scope of the Green's function validation: the analytical reference in Fig. S2 addresses the passive/linear cable equation. For active conductances, there is no closed-form reference, and validation falls back to cross-simulator agreement. I suggest a sentence along the following lines to prevent readers from over-generalizing the passive-case result:

"the analytical validation (Fig S2) confirms solver correctness in the passive/linear regime, where a closed-form reference exists. For models with active conductances, there is no such reference, so agreement is instead assessed via cross-simulator comparison (Figs 2-3), and the residual RMSE observed there is consistent with first-order linearization error that is expected to be largest during fast nonlinear transients such as the spike upstroke."

2. Calibration of the "~10 Hz" memory-formation claim (line 251): the abstract appropriately presents the WTA result as a proof-of-concept; the corresponding claim in Results should be phrased in the same way, since it reflects a single-run rate estimate under explicitly non-physiological stimulation parameters rather than an experimentally derived and validated quantity.

Open Access This Peer Review File is licensed under a Creative Commons Attribution 4.0 International License, which permits use, sharing, adaptation, distribution and reproduction in any medium or format, as long as you give appropriate credit to the original author(s) and the source, provide a link to the Creative Commons license, and indicate if changes were made.
In cases where reviewers are anonymous, credit should be given to 'Anonymous Referee' and the source.
The images or other third party material in this Peer Review File are included in the article's Creative Commons license, unless indicated otherwise in a credit line to the material. If material is not included in the article's Creative Commons license and your intended use is not permitted by statutory regulation or exceeds the permitted use, you will need to obtain permission directly from the copyright holder.
To view a copy of this license, visit https://creativecommons.org/licenses/by/4.0/

Reviewer #1 (Remarks to the Author):

Overall

This work represents a solid technical achievement that addresses a genuine computational need in neuroscience, but suffers from some gaps in positioning and evaluation gaps that undermine its impact.

The technical implementation is well-executed. The NESTML-to-C++ compilation pipeline is elegant, the vectorization approach is efficient, and the integration with NEST's parallel infrastructure leverages existing optimizations effectively. The work is biologically well-motivated, as dendritic calcium dynamics and apical amplification are critical to neural information processing. The Ca-AdEx model example demonstrates meaningful differences between compartmental and point-neuron learning.

The software architecture appears robust and the scaling performance within the framework is impressive.

The manuscript's major weakness is the somewhat outdated competitive ecosystem. By comparing only against NEURON while ignoring e.g. PyTorch-based spiking frameworks such as snnTorch, Norse, SpykeTorch and differentiable ODE approaches, the manuscript positions this work against 2010-era alternatives rather than contemporary solutions.

The NEURON comparison, while showing real performance gains, tests only simple scenarios that may not reflect realistic model complexity where NEURON's flexibility become relevant. Comparisons were not performed against CoreNEURON, which is much more optimized than standard NEURON. The lack of complex synaptic models in the benchmarks is particularly puzzling.

The validation scope is too narrow. One biological example, however compelling, doesn't demonstrate the framework's breadth or establish when researchers should choose the NEST/NESTML approach over alternatives.

The biological insights about dendritic learning are valuable and could stand more prominently as a contribution alongside the technical implementation.

I suggest the following modifications to the manuscript and related computational experiments:

- Acknowledge modern alternatives and position this work as advancing compartmental modeling specifically within NEST, not solving a general field-wide gap
We do consider a CoreNEURON benchmark to be very relevant, and now provide a direct comparison with this in our manuscript. We additionally

005/05.15_el

provide comparisons with Brian 2. We also more clearly outline the use-cases where users should opt for NEST over these alternatives. Finally, we have now significantly extended our set of benchmarks with biophysically realistic neuron models from the Blue Brain Project database, and have additionally implemented our network benchmark in NEURON/ CoreNEURON. On all these benchmarks, we demonstrate significant computational efficiency gains. Together, the expanded CoreNEURON, Brian2, realistic-cell and network benchmarks now present a substantially stronger case for the framework, as our NEST based framework is now clearly demonstrated to provide a highly efficient and scalable CPU-based route for simulating networks of morphologically extended neurons. This justifies our original claim.

- Include at least one comparison against a spiking neuron implementation based on auto-differentiation framework

In this context, it is important to note that although the cited simulation tools implement spiking neurons, they provide no high-level functionality to implement morphological / multi-compartment neurons. Without such functionality, implementing dendritic computation essentially amounts to re-implementing the full tool stack to simulate such models, which is outside of the scope of this work. There is, however, one alternative where compartmental simulation algorithms are implemented in the auto-differentiation framework Jax, in the form of the Jaxley simulator. However, by their own measurements CPU simulations are on par with NEURON, so we consider it unnecessary to implement a direct comparison since we already provide direct comparison with NEURON. In our view, this clearly outlines the use-case for the NEST-based framework as one where scaling is most important — e.g. large-scale brain network studies or networks leveraging local learning — opposed to ease of parameter tuning or learning with e.g. backprop through time. We have now added a discussion of this in the discussion section of our revised manuscript.

- Demonstrate framework capabilities beyond calcium dynamics with additional biological examples

We extend our benchmarks with biophysical models from the Blue Brain Project, which were derived from realistic cells. By consequence, we can now show runtime benchmarks with biophysically realistic cells featuring many ion channels and compartments.

- Include complex synaptic models and CoreNEURON comparisons or acknowledge the limitations of current benchmarks

Cf. our replies to the previous points, we have now addressed this weakness.

- Clearly state when researchers should choose the NEST/NESTML approach versus alternatives

We agree that this should have been outlined more clearly. Our NEST-based approach implements compartmental neuron models in a fully

005/05.15_el

customisable and light-weight fashion. These neuron models are embedded in an extremely scalable simulation framework, where communication across MPI ranks and OpenMP threads is highly optimised, and scales from laptops to supercomputing clusters. Therefore, our framework is suitable for researchers who seek to perform large-scale network simulations, using biophysical learning processes, but have no direct need of auto-differentiation frameworks for gradient optimisation. We have now made this clear in both the introduction and discussion sections.

Following are my detailed comments per section:

1. Introduction

- Clearly articulates the computational bottleneck between detailed single-neuron simulators (NEURON) and large-scale point-neuron networks (NEST)

- The approach of leveraging NESTML within NEST's parallel framework is well-motivated and appears novel

- Reasonable positioning between existing extremes -- more detailed than point neurons but more scalable than full biophysical models; however, a number of solutions for this already exist, such as the Brian simulator.

- Could better quantify the performance limitations of existing approaches

- More expanded comparison of computational trade-offs would strengthen the gap argument

2. Methods

While using implicit Euler for the membrane voltage equation is well-justified for stiff systems typical in biophysical neuronal modeling, and the use of the Hines algorithm leads to really efficient compartmental tree solving, there are some concerning issues with the numerical integration method as described.

First, the scheme makes several approximations that interact problematically:

- Voltage linearization (eq. 3): F is linearized around v(t)
- Leap-frog assumption: State variables see voltage as constant during [t, t+h]

- "Exponential Euler exactness": The claim of "analytical integration" is misleading -- exponential Euler is a semi-analytical method that is only exact given the constant-voltage assumption. But during rapid spiking,

voltage can change by ~100 mV in 1-2 ms, severely violating this assumption.

There are temporal inconsistencies in the order of operations:

- Voltage uses implicit Euler at discrete times: v(t), v(t+h)
- State variables are evaluated at staggered half-steps: y(t+h/2)
- The "analytical" integration of state variables assumes piecewise-constant voltage, which creates a half-step phase lag between voltage and conductances

During fast events (spike upstroke), this staggered timing could produce delayed channel activation relative to voltage, and therefore a broadening of action potentials and incorrect recovery dynamics purely as a result of numerical integration artifacts.

Furthermore, equation (3) linearizes F around v(t), and equation (4) appears to solve once. Is there Newton iteration to refine the solution? For strongly non-linear channels, such as sodium, single-step linearization will be very inaccurate, and the inaccuracies will compound at each spike. How is the Jacobian J_{F} computed?

The use of mixed-order methods is not really justified or validated. Voltage is solved with 1st order implicit Euler, state variables are solved with "exponential Euler" (2nd order if voltage truly constant), or RK5 (5th order for complex cases). The overall error is thus dominated by the 1st-order voltage integration, which means the extra steps of RK5 are somewhat wasteful. More importantly, users may be misled by "5th order Runge-Kutta" into thinking they have high accuracy when the coupling structure limits the overall accuracy.
We acknowledge that our original description of the numerical algorithm creates some confusion, which we clarify in the revised manuscript. It should be noted that the core numerical scheme is simply the gold-standard, which is also implemented in NEURON (when the 'cnexp' is chosen for integration) and in Brian2's SpatialNeuron when the integration method is 'exponential_euler'. We have now clarified that these choices are standard in simulating dendritic neurons and are appropriate when the channel equations are 'conditionally linear', which means that the equations are linear in e.g. variables like 'm', 'h', or 'n' once the voltage-dependent rate-functions being a constant over the time-step are factored in. The 5th order Runge-Kutta scheme is simply NESTML's fallback if this conditional linearity assumption is violated. We have now made this clear in the description of the numerical method.

Finally, we note that indeed, fixed-timestep schemes are of limited accuracy for computing action potential shapes. However, variable time-step schemes would be catastrophic for computational efficiency in distributed network simulations, as nearly all the time there will be a neuron spiking on one of the MPI ranks and/or threads, which will cause all other ranks to

wait. For this reason, network oriented simulators should not use variable time-step integration. Case in point, CoreNEURON also uses fixed timestep integration. We now highlight this limitation, and also why fixed-timestep is the right choice for the use-case considered here.

Recommendations

- Clarify "exponential Euler": explain it's exact only under constant-voltage assumption

We agree with the reviewer and have added a clarification of this to the manuscript.
We note here that to the best of our knowledge, the outlined numerical scheme is closely related to the fixed time-step scheme in NEURON — this is also the only option in CoreNEURON — where the states are solved with `cnexp`, i.e. in the nmodl language there would be a statement of the form `SOLVE states METHOD cnexp`. Such fixed-timestep schemes are also the only admissible option for large network simulations, as advanced techniques backtracking errors during e.g. AP generation would lead to a substantial global slowdown of the entire network simulation. I.e. most CPUs would spend their time idling, waiting for global barrier synchronisation because in a large enough network, there is almost always at least one neuron firing. For this reason, our implementation as well as CoreNEURON accept some potential AP shape inaccuracy in favour of network efficiency. We are also more explicit about the 5th order Runge-Kutta solver, as this is the standard fallback option in ODEToolbox when the equations are not suitable for exponential Euler.

- Add validation section: Test different timesteps, show convergence tests and comparisons with high-accuracy reference solutions

We now test error and spiking accuracy as a function of the timestep explicitly in the revised manuscript.

- Discuss limitations: be explicit about when this scheme may introduce non-negligible errors (for instance when simulating interneurons with high firing rates)

We now explicitly outline this limitation of our numerical scheme, and explicitly describe the motivation (cf. our reply above) to limit ourselves to fixed time steps (same as e.g. CoreNEURON).

- Error estimation: Provide tools or guidelines for users to assess numerical accuracy

W.r.t. to this, we now provide an explicit reference to NEST-NEAT, our NEuronal Analysis Toolkit that provides cross-simulator capability with NEURON. In this way identical models can be simulated in both simulators and, in particular, variable time-step AP simulations in NEURON can be compared with fixed time step ones in NEST/CoreNEURON/Brian2 to empirically assess errors. Note that in the revised manuscript, we compare different timestep widths in both NEURON and NEST, and find similar error magnitude as a function of time step length.

3. Results

- The NESTML to C++ compilation pathway is well-explained and technically sound; 8 compartments per CPU through vectorization shows good optimization

- Strong scaling results (Fig 2F) demonstrate good parallel efficiency

- Ca-AdEx combining abstract and detailed dynamics is innovative

However there are some critical gaps:

- Comparing only to NEURON ignores CoreNEURON and modern PyTorch-based and JAX-based frameworks (snnTorch, Norse, SpikingJelly, Rockpool, etc.)

- There is no comparison to GPU-accelerated spiking frameworks

- The only somewhat biophysical example is Ca-AdEx model with BAC firing

- Single-shot learning is interesting but doesn't validate framework breadth

- There is no comparison across different neuron types, channel types, or network architectures. One example simply doesn't establish broad utility.

- The performance claims are ultimately unclear: without modern baselines, it unclear if this enables previously impossible simulations / research questions.

The manuscript should at least be able to answer the following questions:

- How does the NEST/NESTML framework perform vs. the PyTorch spiking simulation ecosystem or the Jones & Kording-style ODE approaches in JAX?

- How does the NEST/NESTML framework perform with GPU acceleration?

- There were limited morphologies tested: only star and linear dendrites. How do real morphologies behave?

- Some other differentiable biophysical modeling approaches to consider and compare against:

- Lohoff et al. "SNNAX -- Spiking Neural Networks in JAX" (arXiv:2409.02842, 2024)

005/05.15_el

- Heckel & Nowotny "Spyx: A Library for Just-In-Time Compiled Optimization of Spiking Neural Networks" (arXiv:2402.18994, 2024)
- Confavreux et al. "Meta-learning biologically plausible plasticity rules with random feedback pathways" Nat. Comm. (2023)

- Very limited single neuron benchmarks (Fig 2A-D):
- Na+ and K+ channels (Hodgkin-Huxley type)
- No synaptic models: these are purely intrinsic membrane dynamics
- NEURON vs NEST: Fair comparison for channel kinetics only

- Limited network benchmarks (Fig 2E-F):
- Synapses present but unspecified? Networks have connections but no details on synaptic model complexity.
- No NEURON comparison: Only NEST scaling, not NEST vs NEURON for network synapses
- Very basic connectivity: Fixed in-degree, random connections

- Synaptic Model Validation (Fig 3D):
- NEST vs NEST only: Compares standard NEST STDP with their NESTML implementation
- No NEURON involvement: There isn't a NEURON comparison at all

A fair comparison would need:

1. Complex synaptic models: AMPA, NMDA, GABA with realistic kinetics
2. Multiple receptor types: Testing computational overhead of diverse synaptic mechanisms
3. Plasticity rules: Comparing equivalent plasticity implementations across both simulators
4. Realistic connectivity: Testing performance with biologically realistic synaptic densities

Recommendations

- Expand competitive comparisons vs. CoreNEURON, PyTorch-based, JAX-based, other differentiable approaches

As outlined earlier, we now compare all our benchmark neurons also to the CoreNEURON simulator, and a more limited set of benchmarks to Brain 2. Finally, we point to Jaxley as a simulator with automatic gradient computation, which was benchmarked by its creators and found to be on par with NEURON. However, providing equivalent model implementations for every simulator is outside of the scope of this work. We therefore consider our set of benchmarks to be adequate and don't test Jaxley directly.

- Include GPU-accelerated framework comparisons

We are clear and explicit about our approach being constrained to CPU at present. Therefore, to achieve a like-for-like comparison, we focus on CPU performance of the simulators.

- Clearly answer: when should researchers choose NEST/NESTML over alternatives?

We acknowledge that this was not fully clear from our original results, as we only a limited set of direct comparisons. Now, through substantial work crafting a rigorous further benchmark, we can confidently state that our results generalise to biophysically realistic neuron models (Fig 3 in the revised manuscript) and distributed network simulations (Fig 4 in the revised manuscript), for which we now provide direct comparisons against NEURON and CoreNEURON. This makes the use case clear: for developing large-scale, distributed network simulations on CPU clusters (although a NEST-GPU implementation is under development), our NEAT/NESTML/NEST toolchain is highly suited to simulate large distributed networks of morphologically extended neurons.

- Test diverse receptor kinetics: AMPA, NMDA, GABA with realistic multi-state models, short-term plasticity, fair NEURON comparison with long-term plasticity

It should be noted that the benchmarked models contain realistic AMPA, NMDA, and GABA kinetics. We have made this clear in the revised manuscript. Although implementable through the NESTML modelling language in principle, we so far did not test multi-state modelling kinetics. However, because of our vectorised and efficient numerics, there is no reason to assume that multi-state schemes would induce disproportionate performance degradation compared to NEURON. We nevertheless acknowledge this limitation in our revised manuscript.

- Beyond the single Ca-AdEx example, demonstrate or discuss multiple neuron types, fast-spiking, adapting, etc.

We have added a larger set of morphological and electrical neuron models to our comparisons, featuring realistic morphologies as well as a broad variety of channel types.

- Test or at least discuss diverse channel combinations, realistic morphologies, various connectivity patterns, synaptic densities

Cf. the reply above.

- Test performance across a range of firing regimes, e.g. sparse vs. high-frequency activity

The firing rates of our network are on the order of 1-2 Hz for excitatory neurons, and on the order of 8-10 Hz for inhibitory neurons. Although some populations may spike at higher rates or activity may be higher in specific regimes, this is not an order-of-magnitude difference. Further, communication of spikes across distributed networks has many times been proven to be a central strength of NEST, to which we now explicitly refer in the introduction, so in our opinion this is not an aspect that should be

005/05.15_el

retested extensively in the current work, as we do not modify this communication kernel in any way.

- Address numerical accuracy for different regimes: e.g. timestep requirements for regular-spiking, fast-spiking neurons, different morphological complexities

Generally speaking, the time-scales of the biophysical channel mechanisms and intradendritic dynamics are well described, and because of global network synchronisation fixed time step approaches are the only scalable option. We therefore generally fix the time-step of neurons .1 ms, and observe that this captures and stably models spiking properties from fask-spiking to rapid adaptation. In the revised manuscript, we now included fast-spiking interneurons in our benchmark set, and have included an explicit description of the fixed-timestep limitation. In this regard, we also mention CoreNEURON, which also uses fixed-timestep integration for exactly the same reason.

Without these additions, readers cannot assess whether this work represents a meaningful advance over existing solutions or enables previously intractable research questions.

05/05.15_el

Reviewer #2 (Remarks to the Author):

Thank you for this interesting, timely and well-written paper. Frameworks for simulating large networks of 'few-compartmental' neurons are very much needed in the field of computational neuroscience and spiking ML. Furthermore, the vectorising of compartmental updates is both a good ideas and, I imagine, a pragmatic way to exploit extra parallelism without any changes deeper in NEST itself...

My only major criticism of this paper is that it really should compare against Dendrify which aims to solve the exact same problem but builds on the Brian2 simulator rather than NEST. From my understanding of Brian2 and Dendrify, I SUSPECT the equations it generates will be solved using a standard Brian2 solver rather than the Hines method so, it will scale less well with more compartments but, nonetheless, I think figure 2 B,C and D should be extended to include a comparison against Dendrify. I also BELIEVE Dendrify is compatible with Brian2CUDA for GPU simulation.

We explored Dendrify as requested, but found it unsuitable for the present benchmark class because it is based on Brian 2 `NeuronGroups` rather than `SpatialNeuron`. As a result, the coupled compartmental dynamics are advanced by standard forward ODE solvers rather than a Hines-style cable solver. For several of the tree layouts used in our benchmarks, this led either to numerical instability or to timestep requirements that defeated the purpose of the runtime comparison. We therefore used Brian 2 `SpatialNeuron`, which implements the appropriate solver class for arbitrary multicompartment morphologies, as the Brian-based baseline. We have added a paragraph in the discussion pertaining to our choice of benchmark simulators. As GPU implementations are outside of the scope of the current paper (although a NEST-GPU code generation pipeline is in the works), we also constrain our comparison to CPU-only implementations.

Other than that, just a few minor things:

* Line 36 - I think it would be good to be explicit that you are talking about attention here *We made this explicit now*
* Line 73 - I assume this 8-way parallelism is for double-precision float on an AVX-512 CPU. It would be good to make this explicit.

We are now explicit about this.

* Line 85 - STDP

fixed

* Line 89 - There's a stray uppercase V in equation 1 which I think should be lower case. Also I think it would be good to use different parentheses for 'functions' e.g. i_c^i and actual bracketing e.g. around g^j - v^i in the

005/05.15_el

passive system. Finally, I don't think you ever define S_r - presumably it's a weighted sum of incoming spikes.

We now fixed define S_r explicitly and changed the upper case V in lower case. To make notations more clear, we now use square brackets for functions and round brackets for algebraic grouping or time-dependence.

* Line 104 - would be nice to link back to the equation here and repeat that active currents are i_c^i and i_r^i.

We now link back to these quantities.

* Line 105 - "allowing modern CPUs TO integrate"

Fixed

* Line 140-152 - How come this model is constructed to fire at such a low firing rate (1-2spike/second)? That is definitely going to help with being "distributed over many CPUs with little overhead"

It can be noted that even though the exc neurons fire at 1-2 Hz, the inh neurons fire at 5-10 Hz. While these rates are not overly high, they are within reasonable physiological ranges. We now explicitly show the firing rates of the inh neurons as well in the revised manuscript. Further, NEST has been shown in many benchmarks to scale well using point neurons, and therefore will also scale well with multi compartment neurons, since the neuron update is the only part that becomes more complex in this case, and this does not induce extra communication overheads in our setup. We now explicitly point to the fact that NEST is already very efficient in communicating spike times in the introduction of the revised manuscript.

* Line 173 - I assume this is referring to e-prop. It would be good to cite Bellec et al. here. *That is correct, we have added the citation.*

* Figure 3 - I think the font used for the code is WAY too small here. Maybe re-arrange the panels so the code flows across two columns or something. Also, in panel C, how come the dendritic and somatic stimulus currents are different shapes?

We have now increased the font size in panel B. The stimulus currents follow the BAC firing protocol from Hay et al. (2011) which used a step current at the soma and a alpha-function waveform in the dendrite. We have now made this explicit in the manuscript.

* Line 206 - Where does this ten frame per/second number come from?

To explain, we added in the manuscript the following text: "In response to an apical 25 ms contextual signal, a first ensemble is formed in 100 ms (red population in the network supporting apical amplification, Fig 6C,D), start time of learning at 5000 ms), immediately followed by the formation of another assembly (yellow population, start time 5100 ms, same panels). This demonstrates that the Ca-driven apical compartment enables formation of new memories at a rate of ~10 Hz."

* Line 230 - The reason GPUs are particularly good at matrix multiplication and not so great at SNN simulation is that matrix multiplication has a very high arithmetic intensity i.e. each matrix tile read from memory gets a lot of maths done to it which is not really the case for SNNs with simple neurons

We agree with the reviewer that the contrast with artificial neural network workloads should be explained more clearly. We have therefore added a sentence to the Discussion noting that dense matrix multiplication has high

arithmetic intensity, whereas spiking network simulations with simple neuron models typically involve lower arithmetic intensity and more irregular memory-access patterns, which helps explain the more modest GPU speedups often observed for SNN simulations.

* Line 236 - 10.1038/srep18854 would be good to cite here as they already explored this a little, finding that "Izhikevich neurons are much simpler than Hodgkin-Huxley neurons, and therefore can be simulated faster both on GPUs and on the CPU, but the relative speedup of the GPU compared to the CPU was ten folds higher for the benchmark which uses Hodgkin-Huxley neurons"

We thank the reviewer for this great catch and suggestion.

* Line 568 - an additional (and more insidious) issue with fast math is that the flush to zero rounding mode it sets tends to be per-thread so messes up other stuff as well. I don't know if that's an issue with NEST as maybe all the fast math code only runs in a worker thread.

This is an interesting insight. We have so far not observed issues suggesting that flush-to-zero behavior leaks into other parts of the code and adversely affects numerical behavior. We have now stated this more explicitly in the methods section where we describe using the fast math option.

005/05.15_el

Reviewer #3 (Remarks to the Author):

This manuscript describes a new framework for constructing and simulating neural network models based on neuronal models that are intermediate in complexity and biological realism between point neuron models and morphologically detailed model neurons. One expected major area of application is modeling the effects of nonlinear dendritic processes and related plasticity phenomena on network dynamics and learning. This is demonstrated directly in the paper by providing a new network model of the modulation of learning by top-down inputs to the distal dendrites of layer 5 neocortical pyramidal neurons.

This works builds heavily on previously developed software infrastructure related to the NEST simulator, but extends existing tools such as the NESTML model description language in clever ways to enable the efficient simulation of morphologically simplified multi-compartmental models with arbitrary biophysical mechanisms and synaptic plasticity. The technical aspects of the implementation are described in sufficient detail to enable the understanding of the underlying concepts, and links to the source code are also provided. A limited set of benchmarks is also included and shows good scaling of the implementation to more neurons, compartments, and CPUs.

My main concern related to the software framework described is that it is not clear to me how easy it would be for an average user to implement their own models based on the available information. The models showcased by this paper were built with the participation of the researchers who are also actively involved in the development of the underlying tools such as NEST and NESTML, and building the models seemed to require not just deep knowledge of the technical details of these tools, but also their further development. This raises at least two questions. First, can users now expect that the latest versions of the necessary software tools will support a wide range of modeling scenarios without requiring further changes to the codebase? And second, is the documentation of the model-building process and the underlying tools themselves sufficiently detailed to enable new users to apply this framework in their own research? In general, is the framework sufficiently mature to support uninitiated users, or is it currently more suitable for those who are already familiar with the technical details of the underlying tools?
We share the valid concern of the reviewer, that the code stack should be sufficiently broad and robust to support a wide variety of use cases in such a manner that they are accessible for uninitiated users. We are confident that this is the case, as our code is built on top of a set of mature software tools, such as NEST and NESTML. Further, our code supports a wide variety of use cases, from simple few-compartment models where individual compartment parameters are set explicitly by the modeller, to complex biophysically realistic dendrite models, as we now demonstrate in

005/05.15_el

the revised manuscript. The latter are supported by the NEAT API. Although NEAT as a toolbox is younger than the NEST and NESTML, it has extensive documentations and published literature describing its workings. This shows that the codebase supports a broad range of use-cases. Further, the NEST-initiative provides support for users through its Github page, user mailing list, various hackathons, and tutorial/workshop sessions at various neuroscience conferences. We have highlighted both the broad range of applications and user support pathways in the discussion of the revised manuscript.

The last part of the Results section presents a network model in which an appropriately chosen pattern of inputs to the dendritic compartments of excitatory neurons leads to the rapid strengthening of connections between these cells. This simulation is clearly a nice demonstration of the capabilities of the modeling framework described in the first part of the paper; however, it is not entirely clear whether the authors also consider these results as a novel proposal regarding the actual mechanisms that mediate the effects of top-down inputs on learning in the neocortex. If this is the intent of the authors, then I think that this part should be significantly extended, and more motivation, analysis and discussion should be provided. For instance, the choice of parameters in the winner-take-all network should be further motivated, especially since some parameter values (including some specific synaptic conductances and time constants) appear to be outside the range that is generally considered to be biologically plausible. In addition, claims about coordinated vs. random firing should be properly supported by appropriate synchrony measures and statistical analyses. Furthermore, claims such as the one that, without dendritic amplification, one-shot learning would require contextual inputs of “unrealistic strength” should also be properly supported by quantitative data and comparison with relevant experimental results. On the other hand, if the sole purpose of this section is to show the applicability of the software framework to this type of question, then this should be explicitly acknowledged, but it may not be necessary to provide all the detailed analysis that I referred to above – although some further discussion of the assumptions and findings in the context of the relevant experimental literature would still be welcome.

We thank the reviewer for requesting the clarification. We added in the manuscript the following text in the discussion: ”We remark that the demonstration obtained through the WTA-States toy-model is not meant to describe a biologically accurate network. Rather, its purpose is to demonstrate the feasibility of levering apical dynamics for flexible and incremental learning in large-scale spiking networks (Tran et al., Neurips, 2025; Maristany et al, Science, 2026).”

Also, we added at line associated Results section “To this purpose, we customize a toy-model that supports the description of a network composed of assemblies of neurons engaged in a winner-take-all dynamics, capable of incremental learning and of entering different brain states (WTA-States, cf. Methods).”

05/05.15_el

Furthermore, we included in the methods section entitled 'Winner-take-all network' the following text: "WTA-States is used for the initial calibration of the cortical layer in a more complete thalamo-cortical plastic spiking model capable of incremental learning and sleep cycles (ThaCo: Capone et al, Sc. Rep., 2019; Golosio et al., PLOS CB, 2021) through the emulation of apical mechanisms. The ThaCo model is currently under extension to leverage the Ca-AdEx two-compartment neural model (Pastorelli et al., Front. Comp. Neurosc., 2025)."

I also have some more specific comments and questions about the manuscript, which are listed below.

1. In the context of the usability of the software by users with varying levels of expertise, some discussion of error handling and error messages in the different components of the software could be useful.

There are broadly speaking two categories of errors that may emerge. Firstly, there are errors in the NESTML equations, and secondly, there are errors that may occur in the model construction phase in Python. The first set of errors is handled in detail by the NESTML interpreter, and adapts the standard error-generation pipelines where necessary. The second set of errors will primarily arise through erroneous indexing and using variable names that are divergent from those in the NESTML code. Each of these categories of errors raises dedicated and informative error messages. We have now added a description of this in the supplementary information in the revised manuscript.

2. It would be useful to include some discussion of the limitations of the current software framework, and whether there are any plans to extend its capabilities.

We think there are three major limitation: the lack of GPU implementation at this point in time (although a code-generation pipeline for NEST-GPU is currently under development), a lack of gradient based parameter tuning, such as currently provided by Jaxley, and a lack of load balancing for computationally diverse cell types. The first point was already discussed in the original manuscript's discussion section. We have now added a discussion of the second and third points to the manuscript.

3. A technical detail that I think is important from the perspective of efficiency: Does NEST take into account the complexity of neuronal models when the cells are distributed across CPUs?

For load balancing, it would indeed be beneficial to distribute the cells according to computational load across the CPUs. Currently, this does not happen and is a limitation, which we now describe in the discussion (cf. our reply to previous point).

4. The examples in the repositories appear to require the NEAT tool, but it is not clear from the manuscript why it is necessary and how it is used in this case.

As NEAT implements capabilities to export dendrite models to multiple simulators (for the purpose of this revision extended with Brian2 and CoreNEURON), we need it support our multi-simulator tests (as otherwise we would essentially re-implement the same functionality). We have now

005/05.15_el

made this clear in the revised manuscript, and also more clearly describe the use case in which user should incorporate NEAT in the network building workflow vs the use case in which they should directly specify model layouts in the PyNEST API.

5. How will the long-term availability and support of both the software tools and the example models be ensured?

The NEST initiative is treated as key neuroscience research infrastructure by the Helmholtz Association and therefore continued long-term support will be available for the codes and models presented here. Support is generally provided through its Github issue tracker and the NEST user mailing list, as well as various hackathons and tutorial session at neuroscience conferences (e.g. this summer at the CNS meeting in Halifax). We have also highlighted this in the revised manuscript.

6. There are several grammatical and spelling errors that should be corrected. For example, there is an unfinished sentence in line 687; “trough” appears instead of “through” multiple times on page 32; the leak reversal potential is listed as 75 mV (rather than -75 mV) in line 581; there are also typos in lines 186, 198, 205, 592, and 692 that should be easy to fix.

We thank the reviewers for this careful and diligent reading and have addressed the spelling issues.

)05/05.15_el

Reviewer #1 (Remarks to the Author):

Overall

I thank the authors for diligently addressing my concerns. The majority of the issues I raised have been substantively addressed.
The expanded benchmarks against CoreNEURON, Brian 2, biophysically realistic Blue Brain Project cells, and network-level NEURON/CoreNEURON comparisons directly answer my concerns about the competitive ecosystem and validation breadth, and the clarification of the numerical scheme and use-case positioning is appropriate. I recommend minor revisions: the clarification items below are requests for more precise language and a few specific clarifications, and should not be considered objections to the core contribution.

Adequately addressed

- The CoreNEURON comparison is now included across single-neuron, realistic-cell, and network benchmarks (Figs 2-4), resolving my main competitive-baseline concern;
- The addition of Brian 2 SpatialNeuron as a baseline, with explicit justification for preferring it over Dendrify, is well handled;
- The new Fig. 3 with five biophysically realistic BBP models containing diverse Na/K/Ca channels and AMPA+NMDA+GABA receptors addresses both the "single biological example" and "diverse neuron/channel types" concerns;
- The network benchmark now includes NEURON/CoreNEURON comparisons rather than NEST-only scaling (Fig. 4);
- The use-case positioning (large-scale distributed CPU networks with biophysical learning) is now stated clearly in the introduction and discussion;
- The timestep-convergence study (Fig. 2G) and inclusion of fast-spiking interneurons appropriately address the requested accuracy-vs-regime analysis;
- The fixed-timestep justification on network-synchronization grounds is reasonable and well argued;
- The minor notation, citation, and clarity items (S_r definition, AVX-512 explicitness, e-prop citation, fast-math discussion, error handling, load-balancing limitation, NEAT usage) are satisfactorily resolved.

Requests for clarification

- The auto-differentiation comparison is declined on a transitive argument (Jaxley ~= NEURON, NEST beats NEURON); this is understandable given that Jaxley appears to be the only cited tool that supports multicompartment morphologies, but relies on cross-paper, cross-hardware numbers rather than a controlled measurement;

We have made this limitation explicit in the revised version of the manuscript (L326-327).

- Multi-state synaptic kinetics and short-term plasticity were not tested; the claim that multi-state schemes would not "induce disproportionate performance degradation" is an unsupported assertion and should be stated explicitly as untested rather than presumed negligible;

We agree that our claim in this regard was based on extrapolation of the observed compartmental models and on the codes that would be produced by NESTML / ODE-toolbox, rather than on evaluation of a stringent benchmark. We have made this limitation explicit in the revised manuscript (L157-160).

- Please clarify whether a single receptor mechanism can mix continuously integrated quantities (e.g., calcium, NMDA Mg block) with event-driven, spike-triggered updates of other quantities, analogous to NEURON's DERIVATIVE/BREAKPOINT/NET_RECEIVE structure;

We thank the reviewer for raising this point. Mechanisms are fully compatible with mixes of continuously integrated state variables and delta-pulse-type signals like spikes (which includes both network connections and the neuron's own spikes). NESTML syntactic elements enabling this functionality include the "input", "update", and "onReceive" blocks, as well as NESTML's convolution mechanisms. Although our codes leverage this functionality, this was not described explicitly in the original manuscript. We have addressed this in the revised version (L107-108).

- Relatedly, please specify the timing semantics of how a spike delivered via S_r interacts with the leap-frog staggered integration of continuous states within a step (i.e., whether event-triggered modifications are applied at the voltage time point or the staggered half-step), as this bears on accuracy for mechanisms coupling calcium, voltage, and spike timing.

We have clarified the order of operations of our model (L580-583), which is first advancing the state variables (t-h/2 -> t+h/2), then integrating the event-triggered step (t+h/2), and finally advancing the voltage given the state variables (t -> t+h).

On the biological framing of the network model (also related to Reviewer 3's comments)

- There is a contradiction in the revised manuscript between its framing and its demonstration: the single-cell benchmarks (Fig. 3) use genuinely biophysically realistic BBP-derived models, but the flagship network demonstration (Fig. 6) is a WTA "toy-model" that the authors now explicitly state is "not meant to describe a biologically accurate network."
- This creates an inconsistency: the abstract and discussion advance mechanistic biological conclusions (single-shot association learning, ~10 Hz memory formation), yet these conclusions rest on a network the authors disclaim as non-biological, with several parameters (e.g. 3000 Hz contextual Poisson, w_stimulus = 4.0 nA) outside physiological ranges;
- A possible constructive way to resolve this contradiction: the NEURON/CoreNEURON test suites include several reduced models derived from realistic biophysical network models, at least one of which could serve as a biologically grounded network demonstration. This would simultaneously strengthen the realism of the flagship result and provide controlled network-level NEURON/CoreNEURON comparison, addressing both concerns at once and reusing the cross-simulator workflow the authors have already built;

- The requested synchrony measures and statistical support for the coordinated-vs-random firing claims were not added and would further strengthen whichever framing the authors choose.

We thank the reviewer for these suggestions, which are clearly interesting and relevant. Nevertheless, we think constructing a biologically constrained network model is outside of the scope of this work. Plausibly constraining, fine-tuning, and then testing such a model for reproduction of biological first- and second-order statistics is a large project that belongs in a separate paper. We think our revision now covers all relevant axes (network scaleability, realistic neurons, simplified models). We do acknowledge, though, that in the original revision there was a friction in the claims, as our biological statement in the abstract about single-shot association learning is only supported by a proof-of-concept model and not a large-scale, fully biologically plausible one. In the revised manuscript, we have weakened this assertion in the abstract, so that it is clear that our network model is rather a show-case for the simplified dendrite-modelling case, while providing a proof-of-concept implementation of single-shot association learning.

On the numerical integration scheme in Methods

I appreciate the authors' clarifications regarding conditional linearity and the exponential-Euler / RK5 fallback, and the new timestep-convergence analysis in Fig. 2G is a valuable addition. The empirical agreement with NEURON across timesteps is reassuring. I'd like to push on one point, though, not to ask for more work for its own sake, but because I think the authors are unusually well-positioned to make a considerable contribution.
My original questions about Newton iteration and the Jacobian, and about the half-step phase lag between voltage and conductances, were largely answered by appeal to NEURON's "cnexp" scheme as the established standard. I note three things about this framing:
First, "matches the gold standard" is a useful sanity check, but as a primary validation strategy it tends to reproduce the reference implementation's behavior, including any of its approximation artifacts, as opposed to independently characterizing the error. If the computational neuroscience field has only one trusted reference, that reference effectively becomes a black box whose accuracy can't be cross-checked. The authors have built a genuinely independent implementation of compartmental integration. This makes them one of the few research groups in a position to provide an independent point of comparison rather than deferring to the first. A small analysis establishing the scheme's accuracy on its own terms (e.g., against a high-accuracy reference solution at very small timestep, which Fig. 2G partly does, and ideally an analytic or near-analytic case) would be more valuable to the community than NEURON-equivalence alone, and would strengthen the manuscript's standing as a reference tool.

Our toolchains involving NEST-NEAT allow for an interesting axis of comparison so far unavailable to morphological neuron solvers, namely that of validation against Green's function solutions computed directly from passive cable models. In the revised manuscript, we have taken one of the Blue Brain Project morphologies (the L5_TTPC2 morphology) and instantiated it as a passive model. For this model, we computed the Green's function using Koch's method, which is available in NEAT, and then validated NEURON and NEST numerical solutions against it. This provides a solver accuracy test that to the best of our knowledge so far has not been available for arbitrary morphologies.

Second, on the specific technical question: to my understanding NEURON's default solver does perform some form of iterative (Newton-type) refinement of the voltage solution rather than a single linearization, so the "cnexp" comparison may not fully address whether a single solve of eq. (4) is adequate for strongly nonlinear channels at the spike upstroke. Relatedly, stiffness issues arising from features such as dendritic spines are precisely the kind of regime where single-step linearization error can matter, and these deserve study by the community beyond what any single simulator's defaults provide. I'm not asking the authors to solve this in full, but only to either describe explicitly how eq. 4 is solved (single solve vs. iteration) and how J_F is computed, or to state plainly that single-step linearization is used and to bound the regime in which it is reliable.

Although NEURON possesses variable time-step solvers, the default is a fixed timestep solver which, for conditionally linear channel/receptor state variable equations defaults to exponential Euler integration (cnexp method). To the best of our knowledge, this default solver also uses implicit Euler for the voltage. However, NEURON can also use higher order fixed-timestep methods such as Crank-Nicholson, although these are known to produce occasional oscillations. For this reason, we actually moved away from a Crank-Nicholson type method, as these oscillations could easily result in spurious spikes in network simulations. Additionally, NEURON also has variable time-step methods (CVode), which contain iterative solvers that could yield higher accuracy for rapidly changing voltages / state variables. Our objective is now explicitly stated to be scalable network simulations (cf. next point), which clarifies also why we focus on providing the simplest stable method in the form of fixed-timestep implicit Euler. Nevertheless, we note that NEAT provides the option to export equivalent models to NEURON as well as NEST, allowing the study of numerical accuracy of fixed vs variable time-step if desired. We have described more explicitly this multi-simulator compatibility aspect, particularly why it motivates us in not providing variable time-step methods, in the revised version of the manuscript (L284-289).

Third, and I raise this constructively, because I think it actually helps the paper: if NEURON is positioned as the accuracy gold standard against which the method is validated, this is in contradiction with the manuscript's central motivation for offering an alternative. The paper's case for NEST/NESTML rests on efficiency and scalability, not on accuracy superiority, and that's entirely legitimate. But it would be worth making explicit that the contribution is a performance-and-scalability advance at _equivalent_ accuracy to the established reference, rather than letting "validated against NEURON" and "an improvement over NEURON" stand side by side without reconciliation. A sentence framing the accuracy claim as parity (within characterized bounds) and the contribution as scalability would resolve this cleanly.

The reviewer is correct in assessing our work as a performance and scalability advance, rather than an improvement in accuracy. In fact, improving accuracy at the cost of computational efficiency runs counter to our central objective, which is making massive scalable network simulations with dendritic compartments attainable. Therefore, our objective was always equivalent accuracy while maximising performance. We agree that our manuscript's contribution can be framed more explicitly in this way, and have added a sentence that makes our design goal explicit (L284-289).

None of this diminishes what is a substantial and carefully executed body of work; these are requests for sharper framing and a modest independent error characterization, both of which I think would raise the manuscript's value as a community resource.

Reviewer #2 (Remarks to the Author):
Thank you for your hard work on this revision - I think your changes have addressed all of my concerns. I can only imagine adding comparisons to two additional simulators was very time-consuming but, in this case, I really do think that it has significantly improved the paper.

Reviewer #3 (Remarks to the Author):

I thank the authors for carefully addressing my earlier comments and clarifying several issues. I have no further comments on the manuscript.

Replies to reviewer comments

1. Scope of the Green's function validation: the analytical reference in Fig. S2 addresses the passive/linear cable equation. For active conductances, there is no closed-form reference, and validation falls back to cross-simulator agreement. I suggest a sentence along the following lines to prevent readers from over-generalizing the passive-case result:

"the analytical validation (Fig S2) confirms solver correctness in the passive/linear regime, where a closed-form reference exists. For models with active conductances, there is no such reference, so agreement is instead assessed via cross-simulator comparison (Figs 2-3), and the residual RMSE observed there is consistent with first-order linearization error that is expected to be largest during fast nonlinear transients such as the spike upstroke."

We have added this sentence to the paragraph discussing the analytical validation: "This analytical validation confirms solver correctness in the passive regime, where a closed-form reference exists. For models with active conductances, there is no such reference, so agreement is instead assessed via cross-simulator comparison (Fig 2, 3). Discretization errors due to the first order linearization (cf. eq. (2)) were found to be consistent across simulators and in line with the expectation that they are largest during fast non-linear transients such as the spike upstroke."

2. Calibration of the "~10 Hz" memory-formation claim (line 251): the abstract appropriately presents the WTA result as a proof-of-concept; the corresponding claim in Results should be phrased in the same way, since it reflects a single-run rate estimate under explicitly non-physiological stimulation parameters rather than an experimentally derived and validated quantity.

We have prepended "in this network model" to the sentence, which now reads: "Thus, in this network model the Ca^{2+}-driven apical compartment enables formation of new memories at a rate of ~10 Hz." In this way, it is now clear that the assertion pertains to this specific proof-of-concept network model.
