# Supplementary material for: A NEST-based framework unlocks massively parallel simulation of networks of multi-compartment neurons with customizable subcellular dynamics
Source: Commun Biol. 2026 Oct 27;9:1411. doi: 10.1038/s42003-026-11157-0 (PMC13714284; doi:10.1038/s42003-026-11157-0)
Supplement: Supplementary file 2 [file 42003_2026_11157_MOESM2_ESM.pdf]

Code concepts

In the new compartmental modelling framework, the coding of a network simulation featuring dendritic compartments broadly speaking consists of three steps: first, the subcellular dynamics are described in NESTML (Fig S4, top), second, the models are built in the PyNEST API (Fig S4, middle), and third, the models are connected to each other, and to the necessary input generators and recording devices (Fig S4, bottom).

Defining ion channels and receptor dynamics. The passive system in eq. (1) is always assumed to be present, and does not need to be explicitly stipulated in NESTML. Rather, the active currents that feed into it are user-described. These currents can read out the compartmental voltage, which in NESTML is described through the `v_comp` keyword. The compartmental code generator interprets two broad categories of currents: channel currents (Fig S4, top left), which are defined as `inlines` tagged with the `@mechanism::channel` tag, and receptor currents (Fig S4, top right), which are `inlines` tagged with the `@mechanism::receptor` tag. The only semantic difference between them is that receptor currents integrate inputs defined in the `input` block. These current types are defined respectively through the `channel` and `receptor` keywords in the `equations` block in NESTML, and associated parameters and state variables are defined respectively in the `parameters` and `state` blocks. Describing the evolution equations for the state variables then follows the normal NESTML syntax[30]. We note that the integration of spiking inputs by receptors can be described through the `kernel` syntax element, as in (Fig S4, top right), or through an explicit `onReceive` statement, as in Fig S5A.

Constructing models in the PyNest API. The currents described in NESTML are the ones that will be available to include in the compartments of the model when it is constructed. At runtime, in the PyNEST API, the model is constructed by initializing its `compartments` attribute from a list of dictionaries, that (i) encapsulate the dendritic tree structure by stipulating a parent index for each compartment, and (ii) that feature a dictionary of parameter values describing both the parameters of the passive system (cf. eq. (1)) and the NESTML-defined channel mechanisms (Fig S4, middle left). To reference the compartments later on, each compartment is assigned an index that corresponds to its position in the compartments list. When certain parameters are not stipulated in the parameter dictionary, the default values defined in the `parameters` block of the NESTML code are used. Note that channel currents are first analysed by the code generator, and will only be simulated if the parameters are such that the current is not zero (e.g. if the maximal conductance is zero, the current will not be simulated in that compartment). In this way, one can flexibly customize the set of currents that will be simulated in each compartment.

After the compartments have been set, receptors are added to the model by setting the `receptors` attribute (Fig S4, middle right). Receptor currents differ from ion channels, in that there can be at most one ion channel of a given type per compartment, but there can be an arbitrary number of receptors. Receptors are also initialized as a list of dictionaries, each individual dictionary featuring the index of the compartment it targets, the receptor type, which corresponds to one of the NESTML-defined names of the receptor currents, and optionally a parameter dictionary. To connect inputs to the receptors, the standard NEST syntax is used (Fig S4, bottom left), where the receptor type corresponds to the index of the targeted receptor in the receptors list. Finally, to record state variables in the model, the standard multimeter is used, where the NESTML-defined name of each state variable is suffixed either by the compartment index – if the state variables is associated to a channel – or the receptor index – if the state variable is associated to a receptor (Fig S4, bottom right).

Error handling and debugging. Errors encountered when building compartmental models in the present framework broadly fall into two classes. First, errors may arise on the NESTML side, for instance through syntactically malformed equations, undefined symbols, or inconsistent use of mechanism annotations. These are handled through the standard NESTML parsing and semantic checking pipeline, which reports the location and nature of the error and can be configured with different verbosity levels to provide more detailed diagnostic output during debugging. Second, errors may arise during model construction in the PyNEST API, for example through invalid compartment indices, inconsistent parent-child relationships in the compartment tree, incorrect receptor indices, unknown parameter names, or invalid recordable names. Such errors are caught during model construction and configuration, where dedicated error messages indicate which part of the Python-side specification is inconsistent with the generated model. In practice, this enables an incremental debugging workflow in which users first validate the NESTML model definition, and subsequently build and test the compartmental structure, receptor configuration, and recording interface step by step.

Integrate-and-fire type mechanisms. To implement integrate-and-fire type mechanisms, users can leverage the NESTML `update` and `onReceive` blocks. The update block allows for the insertion of arbitrary control statements that govern the evolution of the system, but are not part of the smoothly evolving ODEs defined in the `equations` block. This includes, for instance, `if-else` statements that measure threshold crossings. The `self_spikes` variable has a special meaning in NESTML, and signifies the neuron's own spikes. We use it here in the `onReceive` block, to trigger the initiation of the absolute refractory period and the addition of the spike-triggered adaptation (Fig S5A). Accordingly, we use the update block to measure the duration of the absolute refractory period. We note that the ongoing dynamics of the model cannot be paused during the absolute refractory period, as is the case with point neurons, as the dendritic compartments still integrate inputs normally. For this reason, we implement the refractory period as a channel mechanism (`refr` in Fig S5A) that clamps the voltage to the reset value (Fig S5D, top), following the original formulation of the Ca-AdEx model that was directly written in C++ code for NEST[16].

To simulate the AdEx dynamics only at the soma, we provide the desired parameters to the somatic compartment during model construction in the PyNEST API, but set the maximal conductances to zero in the dendritic compartment (Fig S5B), resulting in a model with a passive dendritic compartment and AdEx somatic compartment (Fig S5C). Finally, we illustrate the resulting dynamics by targeting the somatic and dendritic compartment with excitatory and inhibitory Poisson inputs (Fig S5D), confirming (inset) the correct generation of somatic spikes along with the refractory period and reset, decoupled somatic and dendritic dynamics (top), the correct behaviour of the spike-triggered adaptation (middle) and of the exponential threshold current (bottom).

Concentration dynamics. To introduce slow concentration dynamics, equation or set of equations can be marked with the `@mechanism::concentration` tag. In doing so, we effectively instruct the code generator that they can be factored out, and that mechanisms reading the associated variables can effectively consider them to be constant for the duration of an integration step. Without it, the code generator would effectively have to consider the calcium current, potassium current and slow mechanism as a single system of $N_{\text{tot}} = N_{\texttt{chan\_Ca}} + N_{\texttt{chan\_K}} + N_{\texttt{c\_Ca}}$ state variables, requiring e.g. propagation matrices of size N_{tot}^2. Through the use of the `@mechanism::concentration` moniker, the code generator knows that this can be split up in three systems of respective sizes $N_{\texttt{chan\_Ca}}$, $N_{\texttt{chan\_K}}$, and $N_{\texttt{c\_Ca}}$. By extension, the `@mechanism::concentration` keyword allows the efficient incorporation of various processes that proceed on a slow time-scale compared to the fast channel integration, allowing for instance the simulation of various molecular signalling pathways.

Combining plasticity processes and compartments. To implement plastic synapses, several options exist in the NESTML compartmental modelling framework. First, it is a reasonable strategy, especially when the plasticity rule follows a complex interplay of post-synaptic factors, to implement the associated set of equations as part of the compartmental model, leveraging the features described previously. Secondly, one could also create NESTML-defined synapse models, or use existing ones, and compile them together with the compartmental model (Fig S7A, top). This results in the generation of receptor currents that feature the desired plasticity process as part of their dynamics, for every receptor current implemented in the NESTML compartmental model. The name of this receptor current then follows the convention `{receptor name}_{plastic synapse name}_nestml` (Fig S7A, bottom left). When following these two strategies, the connection should be initialized as a static synapse in the PyNEST API, as the plasticity dynamics are simulated entirely on the post-synaptic side. Finally, if the plasticity model is part of the standard model collection in NEST, it can also be specified when the connection is initialized (Fig S7A, bottom right). In this case, the receptor itself should be the static version.

Supplementary figures

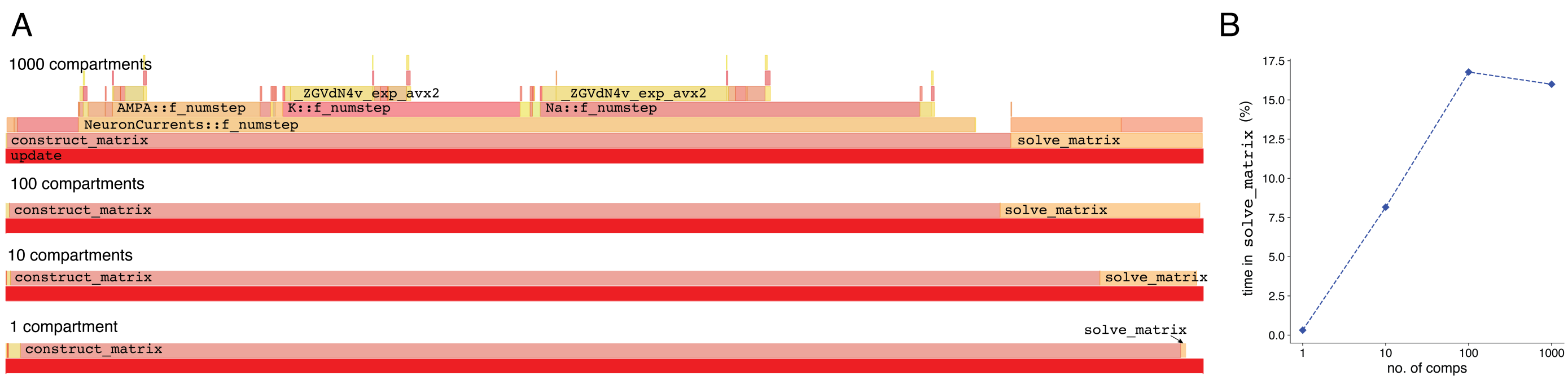

Supplementary Fig. S1 | Profiling results for the MC framework. A: Profiling graphs of the runtime correlate extracted with the Hotspot profiler[1], quantifying the relative fraction of time spent in specific function calls in the linear dendrite model from Fig **??**A. We highlight the `update` function (red), which implements the full update step of the model. This function is broken down in a `construct_matrix` part and a `solve_matrix` part, which respectively construct and solve the Hines matrix. Through the `construct_matrix` function all relevant update functions are called. Here, there are an AMPA receptor, a K channel and a Na channel in each compartment (shown for the 1000 compartment case, top). Decomposing these updates reveals that (i) these functions are vectorized properly as the vector primitive of the exponential function is invoked and (ii) that they spend most of their time evaluating exponentials to compute the channel activations and advance the channel state variables. Profiling results were obtained on a laptop with Intel(R) Core(TM) i5-4210H CPU @ 2.90GHz and 16 GB DDR3 1600MHz Ram **B:** Quantification of the fraction of time spent solving the Hines matrix (i.e. in the `solve_matrix` function) vs. the combined time spent to construct and solve the matrix (i.e. `construct_matrix` + `solve_matrix`) as a function of the number of compartments.

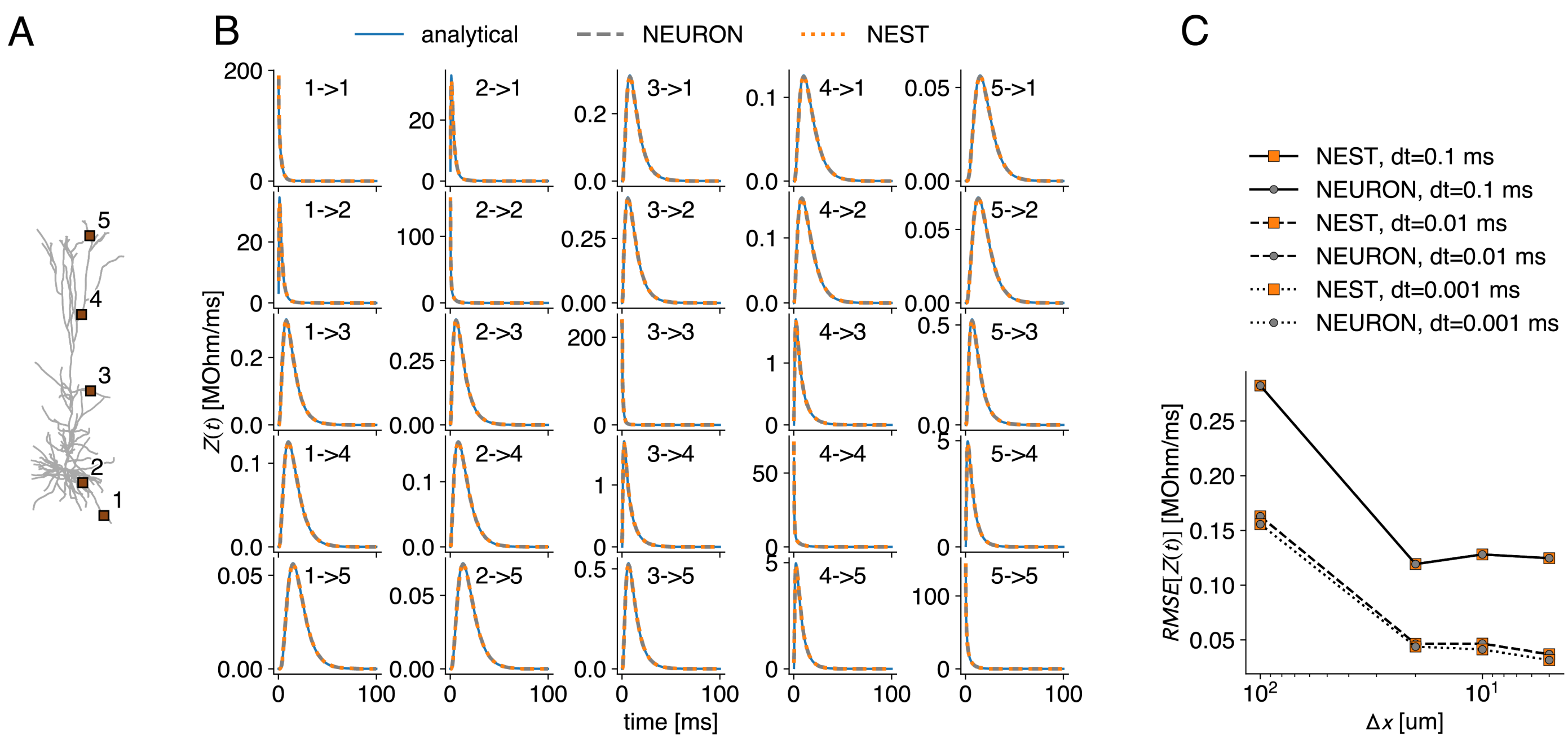

Supplementary Fig. S2 | Comparison with analytical spatio-temporal reference solution. A: L5_TTPC2 morphology with 5 locations, between which we evaluate accuracy of impulse response kernels $Z(t)$ with respect to a reference analytical solution. Note that the error of the spatio-temporal discretization itself is compounded by a spatial resolution error, which is induced by assigning locations to the nearest compartment on the spatial grid. We remove this compounding error by shifting the reference locations for the analytical solution to the mid-point of the nearest compartment. **B:** Impulse response kernels $Z(t)$ between the 5 sites simulated with both NEST (orange) and NEURON (grey), compared to the analytical NEAT solution (blue). Simulations were performed at $dt = 0.1$ ms and $dx = 20$ μm. **C:** RMSE compared to the analytical solution for various time- and distance steps.

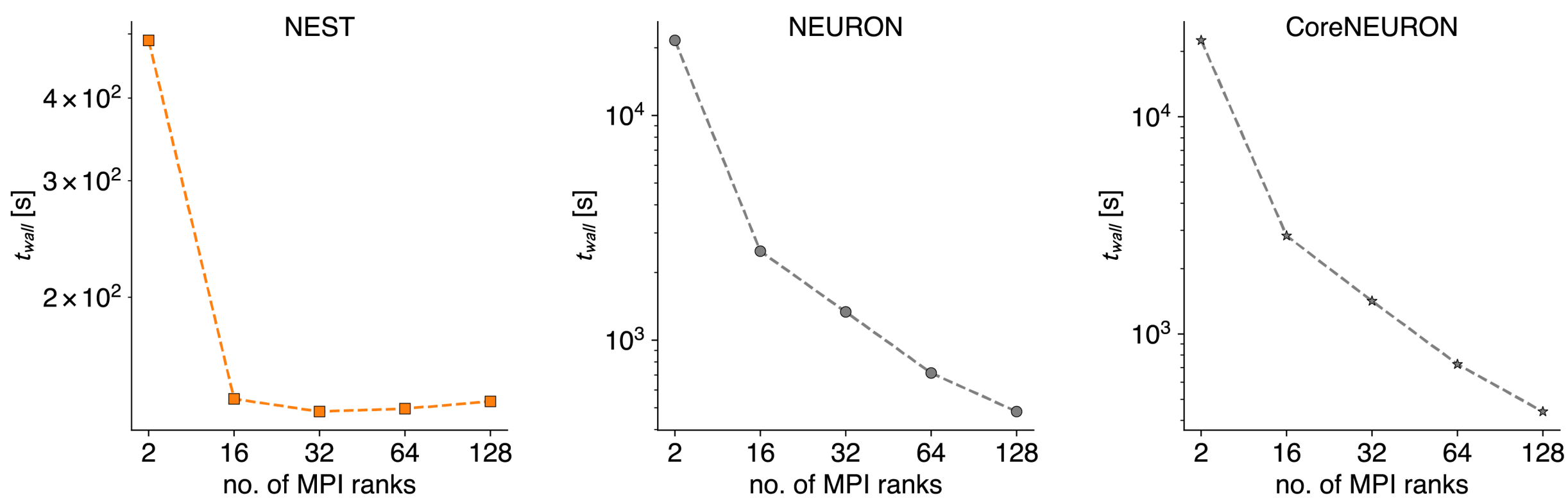

Supplementary Fig. S3 | Optimal MPI/multithreading configuration. Wall clock runtime for the same simulation, distributed across varying numbers of MPI ranks. The number of threads per MPI rank was adjusted according to $\frac{128}{\text{no. of MPI ranks}}$, with 128 the number of CPU cores on the node.

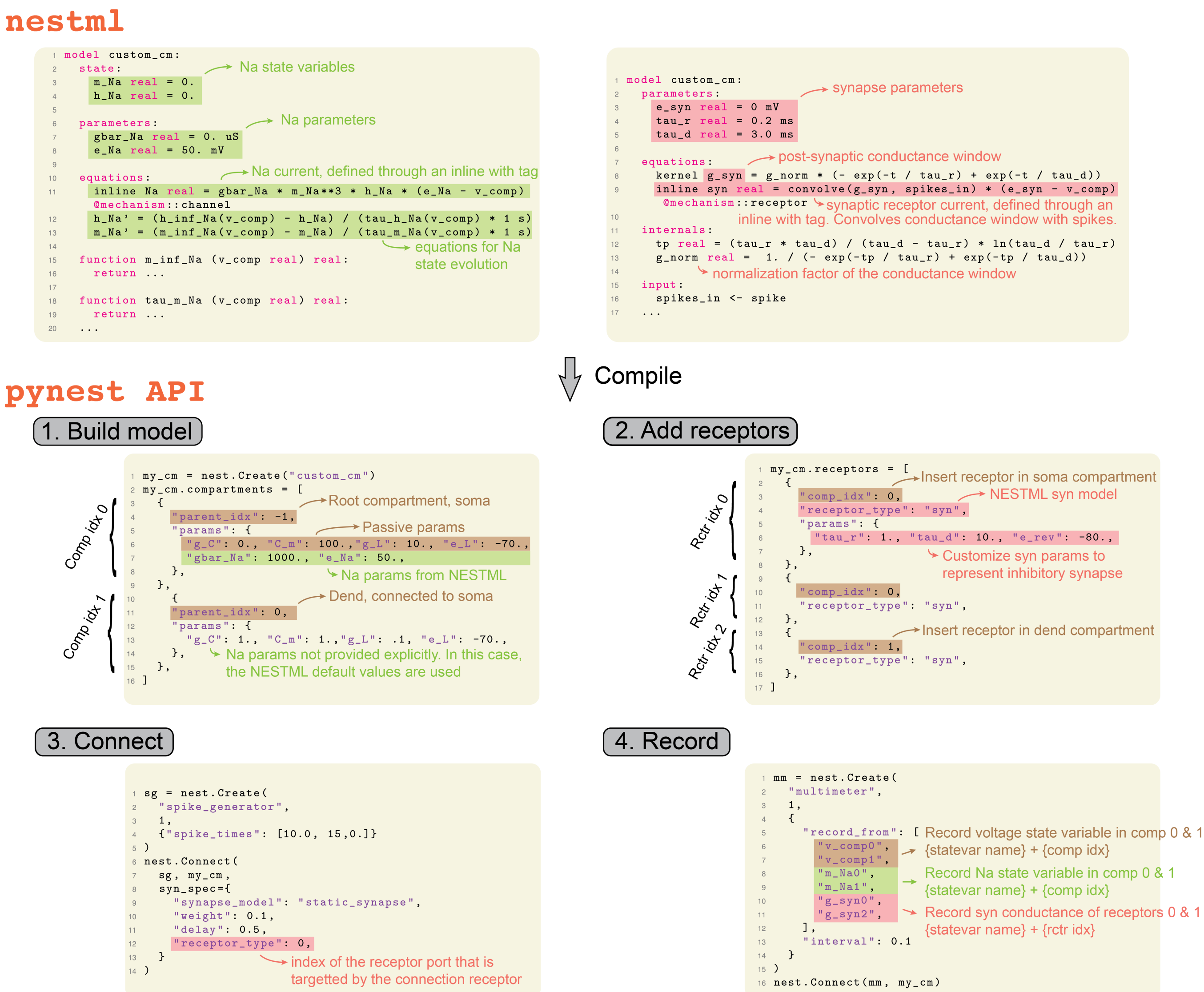

Supplementary Fig. S4 | Basic code concepts in NESTML and the PyNEST API. Ion channel currents (upper left) are defined through `inlines` tagged with the `@mechanism::channel` tag. The current consists of state variables and parameters, which are defined in the respective NESTML blocks. State variable evolution equations follow the standard NESTML syntax. Receptor currents (upper right) are defined in a similar manner as channels, but are tagged with the `@mechanism::receptor` tag, and should integrate some input (here a spiking input, but continuous inputs can also be integrated – see documentation). To build the model in the PyNEST API (middle left), an empty model is initialized with `nest.Create()` and then equipped with compartments. For each compartment, custom parameter values or state variable initializations can be specified in the `"params"` dictionary. The compartments get assigned an index based on their position in the compartments list ('Comp idx' in the figure). Note that the parent compartment (specified with `"parent_idx"`) can only refer to a compartment at a prior position in the `compartments` list, and that the somatic compartment (the 'root' node of the tree) should have `-1` as parent index. After the compartments have been set, the receptors can be added (middle right) by specifying the compartment index that they target, the type of receptor (one of the types defined in NESTML) and the parameters and state variable initializations. Similarly to compartments, receptors get assigned an index corresponding to their position in the `receptors` list ('Rctr idx' in the figure). Finally, the models can be connected (bottom left), specifying the index of the receptor port that a connection targets as part of the `syn_spec` dictionary (the `"receptor_type"` entry), and recordables can be set by connecting a `"multimeter"` to the neuron model (bottom right). Note that any NESTML defined state variables, inlines or kernel convolutions can be recorded.

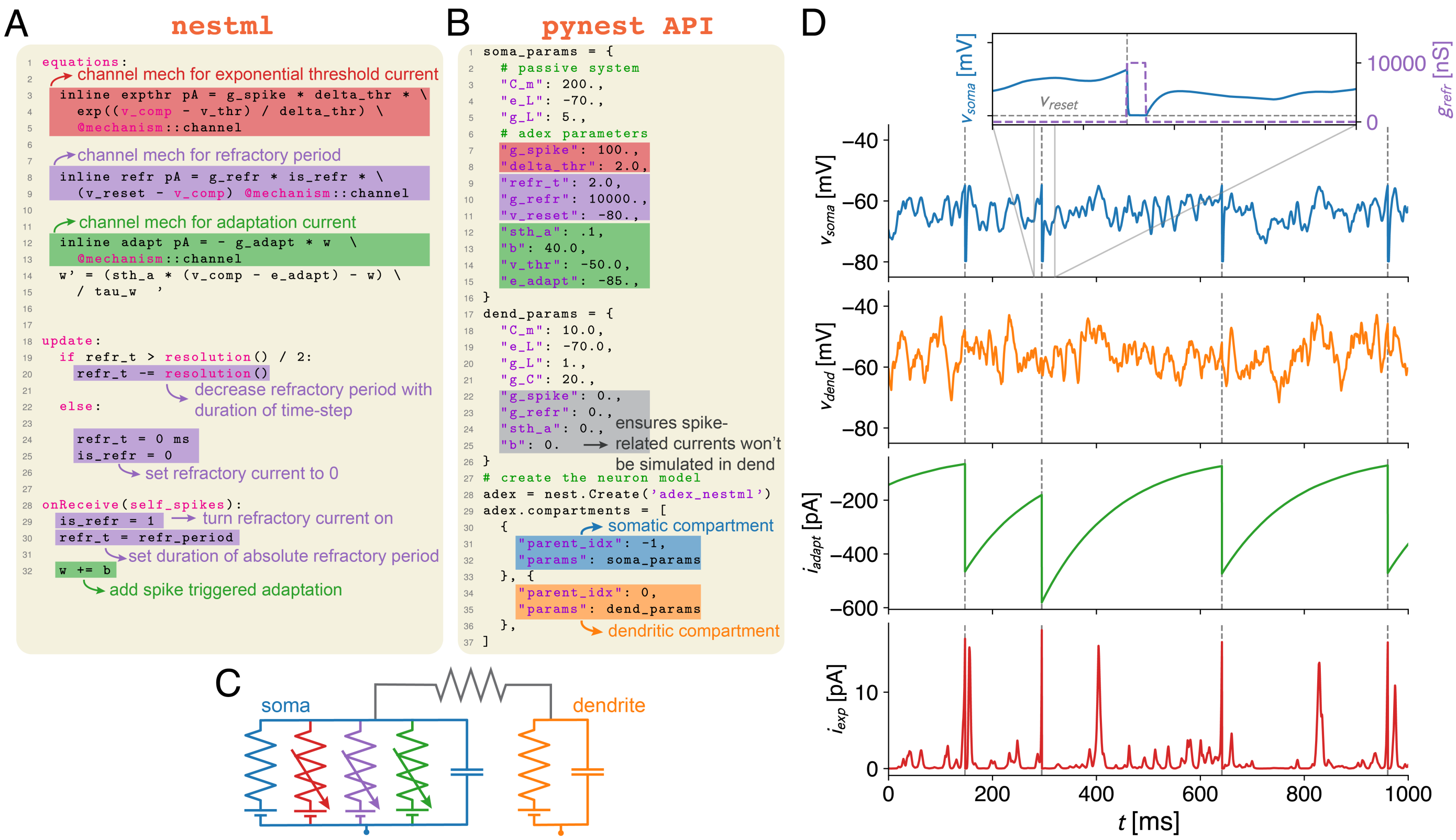

Supplementary Fig. S5 | Implementing adaptive exponential integrate-and-fire (AdEx) dynamics in the compartmental modelling framework. A: The NESTML language allows for a compact description of non-linear trans-membrane current dynamics, here illustrated with the AdEx model. This model features an exponential threshold current (`expthr`, red) and an adaptation current (`adapt`, green). While the spike threshold is set in the PyNest API (B), spike adaptation (green), refractory period and reset (purple) dynamics are implemented on the NESTML side using the `onReceive` and `update` blocks. As compartmental dynamics are always ongoing, the absolute refractory period is implemented by clamping the voltage to the reset potential. Note that these dynamics defined in NESTML represent transmembrane currents that *can* be implemented in each compartment of the eventual model, but whether they *will* be simulated in the compartment depends on the final parameter choice. **B:** At model creation in the PyNest API, compartment parameters are set to their final values (note that parameters not explicitly defined in the `"params"` dictionaries are initialized to their NESTML default values). Here, in the dendritic compartment (orange), conductance parameters for the AdEx currents (`expthr`, `adapt`, and `refr`) are set to zero, while in the somatic compartment (blue) they are set to non-zero values. **C:** Schematic of the resulting model. Note that because the conductance parameters of the AdEx mechanisms were chosen such that these currents are identically zero in the dendritic compartment, they are *not* simulated there. **D:** Exemplar simulation of the resulting model, stimulated with somatic and dendritic Poisson inputs, illustrating (from top to bottom) the somatic voltage with an inset detailing the refractory period implementation, the dendritic voltage, the `adapt` current and the `expthr` current.

```
generate_nest_compartmental_target(
  input_path=["resources/ca_adex.nestml", "resources/stdp_synapse.nestml"],
  ...
  codegen_opts={
      "neuron_synapse_pairs": [
          {"neuron": "ca_adex", "synapse": "stdp_synapse", "post_ports": ["post_spikes"]}
      ],
      "delay_variable": {"stdp_synapse": "d"}, "weight_variable": {"stdp_synapse": "w"},
  },
)
```

NESTML compartmental STDP

```
post_neuron.receptors = [
  {
    "comp_idx": 0,
    "receptor_type": "ampa_nmda_stdp_synapse_nestml",
    "params": {
      "w_stdp": 10.0,
      "Wmax": 20.0,
      "lambda": 0.05,
      "alpha": 1.0,
    },
  }
]
nest.Connect(
  pre_neuron,
  post_neuron,
  "all_to_all",
  syn_spec={
    "synapse_model": "static_synapse",
    "weight": 1.0,
    "receptor_type": 0,
    "delay": sim_cfg.dt
  },
)
```

plastic receptor
STDP params
static synapse with dummy weight

NEST STDP

```
post_neuron.receptors = [
  {
      "comp_idx": 0,
      "receptor_type": "ampa_nmda",
  }
]



nest.Connect(
  pre_neuron,
  post_neuron,
  "all_to_all",
  syn_spec={
    "synapse_model": "stdp_synapse",
    "weight": 10.0,
    "Wmax": 20.0,
    "lambda": 0.05,
    "alpha": 1.0,
    "receptor_type": 0,
    "delay": sim_cfg.dt,
  },
)
```

static receptor
plastic synapse
STDP params

Supplementary Fig. S7 | Adding plasticity synapses to the multi-compartment models. NESTML-defined plasticity rules can be compiled together with a neuron model (top). In this case, the plasticity rule is combined with every receptor current defined in the neuron model. Note that the pure receptor current – without plasticity – is also still available. In the PyNEST API, this plastic receptor is added when initializing the `receptors` list, whereas the connection itself is initialized as being static (bottom left). Standard plasticity rules already defined in NEST can also be used, in this case, the original receptor is added in the `receptors` list, but the connection is initialized as a plastic synapse (bottom right).
